# grenedalf: population genetic statistics for the next generation of pool sequencing

**DOI:** 10.1093/bioinformatics/btae508

**Published:** 2024-08-26

**Authors:** Lucas Czech, Jeffrey P Spence, Moisés Expósito-Alonso

**Affiliations:** Department of Plant Biology, Carnegie Institution for Science, Stanford, CA 94305, United States; Section for GeoGenetics, Globe Institute, University of Copenhagen, 1350 København, Denmark; Department of Genetics, Stanford University, Stanford, CA 94305, United States; Department of Plant Biology, Carnegie Institution for Science, Stanford, CA 94305, United States; Department of Biology, Stanford University, Stanford, CA 94305, United States; Department of Global Ecology, Carnegie Institution for Science, Stanford, CA 94305, United States; Department of Integrative Biology, University of California Berkeley, Berkeley, CA 94720, United States; Howard Hughes Medical Institute, University of California Berkeley, Berkeley, CA 94720, United States

## Abstract

**Summary:**

Pool sequencing is an efficient method for capturing genome-wide allele frequencies from multiple individuals, with broad applications such as studying adaptation in Evolve-and-Resequence experiments, monitoring of genetic diversity in wild populations, and genotype-to-phenotype mapping. Here, we present grenedalf, a command line tool written in C++ that implements common population genetic statistics such as *θ*, Tajima’s *D*, and *F*_ST_ for Pool sequencing. It is orders of magnitude faster than current tools, and is focused on providing usability and scalability, while also offering a plethora of input file formats and convenience options.

**Availability and implementation:**

grenedalf is published under the GPL-3, and freely available at github.com/lczech/grenedalf.

## 1 Introduction and motivation

Pool sequencing, or Pool-seq, is a cost-effective high-throughput sequencing method for obtaining genome-wide allele frequencies across multiple individuals simultaneously (Schlötterer *et al.* 2014). The approach is commonly used in large-scale genomic studies to estimate genetic diversity and variation within a population across space and time, or to identify genetic changes that are associated with trait evolution or environmental adaptation across populations. This makes it suitable for applications such as studying adaptation in Evolve-and-Resequence (E&R) studies, genotype-to-phenotype mapping, or pooled genome scans and mutant screens.

The pooling of a finite number of individuals from the population, as well as the finite number of reads being sequenced from each individual, introduce two levels of sampling noise in allele counts (Ferretti *et al.* 2013). Typical population genetic statistics, such as measures of diversity (*θ*, Tajima’s *D*) and differentiation (*F*_ST_), hence need to be adapted to correct for the induced biases. Existing software tools that implement these corrections are PoPoolation (Kofler *et al.* 2011a,b), poolfstat (Hivert *et al.* 2018, Gautier *et al.* 2022), and npstat (Ferretti *et al.* 2013). These tools however lack usability, do not scale to contemporary large datasets, and do not support haplotype-corrected frequencies in low-coverage E&R experiments such as those from HAF-pipe or other HARP-based pipelines (Kessner *et al.* 2013, Tilk *et al.* 2019).

We present grenedalf, a command line tool to compute widely used population genetic statistics for Pool-seq data. It aims to solve the shortcomings of previous implementations, and is several orders of magnitude faster, scaling to thousands of samples (Czech *et al.* 2022). Further, it improves usability, accepts many standard file formats, and offers many convenience options.

## 2 Estimators of population genetic statistics

We re-implemented consistent estimators of population diversity and differentiation, namely nucleotide diversity $\theta_{\pi }$, Watterson’s *θ*, Tajima’s *D*, and Nei’s and Hudson’s *F*_ST,_ which account for the noises introduced by the two finite sampling processes of individuals and reads in Pool-seq. Several of these estimators were previously available in multiple software packages implemented in Perl (Kofler *et al.* 2011a,b), R (Hivert *et al.* 2018, Gautier *et al.* 2022), or C Ferretti *et al.* (2013). Because of implementation differences of estimates available in these packages, we re-derived population genetic estimates and examined their differences (see Supplementary Material).

Most commonly, our input are sequence reads or read-derived allele counts, as those fully capture the effects of both sources of noise, which can then be corrected for. Our implementation however can also be used with inferred or adjusted allele frequencies as input, for instance using information from the haplotype frequencies of the founder generation in E&R experiments (Kessner *et al.* 2013, Tilk *et al.* 2019). These can elevate the effective coverage, and thus improve the calling of low-frequency alleles, which can otherwise be difficult to distinguish from sequencing errors (Ferretti *et al.* 2013). With these reconstructed allele frequencies, the correction for read depth is less relevant, but the correction for pool size remains important. It is hence convenient to be able to use the same framework for these data, which existing implementations do not offer.

### 2.1 Genetic diversity (*π*)

Our implementation of the Pool-seq estimators for $\theta_{\pi }$ and Watterson’s *θ* largely follows the approach by PoPoolation. We have however updated some of the equations with computationally more efficient but otherwise equivalent alternatives, and have improved the numerical stability.

However, in our attempt to re-derive a Pool-seq estimator for Tajima’s *D*, we noticed several long-standing issues in the existing estimator. In short, it seems that Pool-seq data might not allow meaningful estimates of this statistic; see the Supplementary Material for details. In addition, we noticed several implementation bugs in PoPoolation (Kofler *et al.* 2011a), up until and including v1.2.2 of the tool. We discussed these with the authors, and the bugs have since been fixed (pers. comm. with R. Kofler). If conclusions of studies depend on numerical values of Tajima’s *D* computed with PoPoolation, we recommend reanalyzing the data. Due to these statistical issues, we generally advise to be cautious when applying and interpreting Tajima’s *D* with Pool-seq data.

### 2.2 Population differentiation (*F*_ST_)

We also show that the estimators for *F*_ST_ as implemented in PoPoolation2 (which we call the “Kofler” and “Karlsson” estimators) are biased upward for low read depths and for small sample pool sizes (see Supplementary Material). We hence developed unbiased estimators for the average pairwise diversity within populations, $\pi_{\text{within}}$, the pairwise diversity between populations, $\pi_{\text{between}}$, and the pairwise diversity across the combined populations, $\pi_{\text{total}}$, that take the particular biases and assumptions of Poo-seq data into account. With these, we compute two variants of *F*_ST,_ following Nei (1973) and Hudson *et al.* (1992):

$$F_{\text{ST}}^{\text{Nei}}=1-\frac{\pi_{\text{within}}}{\pi_{\text{total}}}\text{ and }F_{\text{ST}}^{\text{Hudson}}=1-\frac{\pi_{\text{within}}}{\pi_{\text{between}}}$$

We provide thorough derivations and analyses of these estimators in the Supplementary Materials, and showcase their application in Czech *et al.* (2022).

## 3 Features and data processing flow

Beyond implementing a variety of population genetic estimates, our C++ software library and command line tool were designed to address several bioinformatics challenges which limit the next generation of pool-sequencing applications: (i) flexible and modular architecture. Different Pool-seq softwares use different file formats. We separated file format reading and transformations from computations and algorithms. File format transformations are seamless, and new formats can be included independent of downstream analyses. (ii) Usability. Software for large-scale individual genotypes (i.e. VCFtools, PLINK, etc.) provide convenience tools for merging, filtering, and manipulating datasets. We provide these for Pool-seq formats. (iii) Speed. Current tools are too inefficient to allow modern Pool-seq datasets and experiments to grow into hundreds or thousands of population samples. Our highly optimized routines provide orders of magnitude gains in speed.

**Figure 1.**
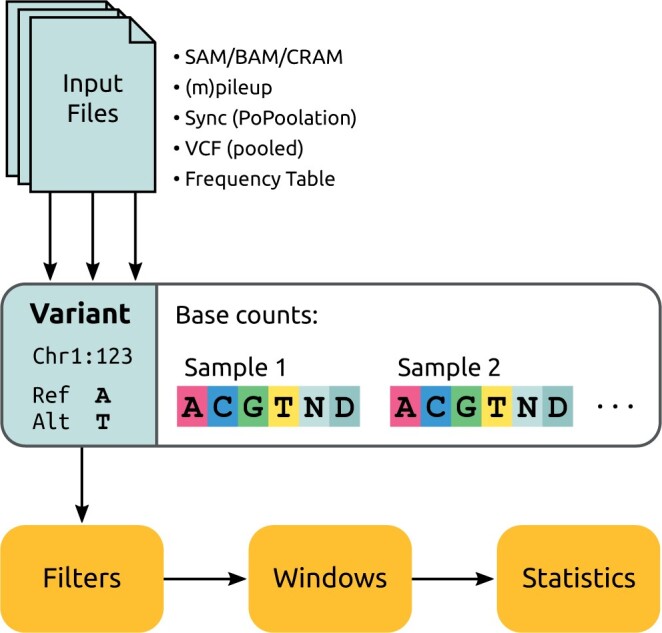
Summary of the data flow from input files to stream through a genome and compute statistics. Different input file types are supported with their idiosyncratic options, which all are represented by a uniform data type that we call a (potential) Variant. A Variant describes a single position on a chromosome, here, position 123 on chromosome Chr1, and stores the reference and alternative base for file formats that support them (and otherwise infers them from the two most common bases at the position, or from a provided reference genome file). This is similar to the data of the sync format. For each sample of the input (e.g. read groups in SAM files, columns in mpileup files, or sample frequencies from tabular formats), the nucleotide base counts (ACGT) of the pooled reads are stored, including counts for “any” (N) and “deletion” (D), which are however ignored in most statistics. The stream of Variants along the genome is then filtered using a cascade of filters, such as sub-setting to regions of interest and numerical quality filters. Next, the data stream can be assembled into different types of windows, such as sliding windows, single positions, or entire chromosomes. Finally, the desired statistics are computed per window.

In the following, we provide an overview of the data processing flow, which is summarized in Fig. 1.

### 3.1 File formats

Two commonly used file formats for Pool-seq data are the (m)pileup (Li *et al.* 2009) and sync format. The latter is a simple allele count format introduced in PoPoolation2 (Kofler *et al.* 2011b), which is usually obtained by converting from bam via (m)pileup to sync, requiring an additional data transformation step to analyze the data.

In contrast, and in addition to these formats, grenedalf can directly work with other standard file formats such as sam/bam (Li *et al.* 2009), cram (Fritz *et al.* 2011), vcf (using the “AD” allelic depth field) (Danecek *et al.* 2011), and a variety of simple table formats, for reading allele counts or allele frequencies from pool sequencing data. All formats can also optionally be gzipped (decompression is done asynchronously for speed), and their idiosyncratic options (such as filtering by read flags or splitting by read groups for sam/bam) are supported. This eliminates the need for intermediate file conversions, reduces overhead for file bookkeeping, disk space, and processing time (see Supplementary Material), and increases user convenience.

Note that not all data types are well suited for the Pool-seq approach. For instance, a widespread practice is to use a variant calling tool on the data before downstream analyses. However, many standard variant callers were developed for individual instead of pooled data, meaning that their statistical assumptions might be violated in Pool-seq (Czech *et al.* 2022). Furthermore, formats such as vcf only store variant sites in the first place, so one cannot distinguish if a missing site is invariant or did not meet the minimum data quality threshold. Mask files as explained below can be used to remedy this. We still support vcf as a convenience, but recommend to ensure that the variant calling was conducted appropriately for the Pool-seq approach. For this reason, it is often beneficial to directly work off the “raw” data, such as sam/bam files or sync files that were not already filtered for SNPs, when running grenedalf.

If a reference genome is provided, it is used to fill in the reference bases when using file formats that do not store these. When multiple input files are provided (even of different formats, and with missing data), they are traversed in parallel, using either the intersection or the union of the genomic positions present in the files, and internally combined as if they were one file with multiple samples. Samples can furthermore be grouped by merging their counts, for instance to combine different sequencing runs into an (artificial) pool.

### 3.2 Filters

After parsing the input, a variety of filters can be applied to the data stream, either per-sample or across samples. First, we can apply sample sub-setting, and sub-setting to chromosomes or genomic regions within or across chromosomes, using a variety of formats [bed, GFF2/GFF3/GTF, map/bim (PLINK), vcf, or simple text formats]. The region filters completely remove genomic positions from the data stream, to speed up the downstream steps.

Next, masks can be specified, in order to pre-select loci of interest. This is for instance useful when an external filtering was applied to the data beforehand; the masks then specify which positions were considered “valid” by that filter. This is important in order to correctly compute the per-window averages of the statistical estimators, where we need to know the number of high-quality loci (independently of whether they are SNPs or invariant positions). Masks can be provided either per sample, or one mask for all samples.

Lastly, users can specify a variety of numerical quality filters, such as minimum allele count and minimum or maximum read depth. Some of the numerical filter settings are highly relevant for the computed estimators; see the Supplementary Material for details. Some commands then offer to sub-sample or re-scale the counts to limit excessively high read depths.

After all specified filters have been applied, we execute a simple SNP detection, based on the base counts (ACGT). Any locus that has (after filtering) exactly one base with nonzero count is considered invariant, while a locus with two or more nonzero counts is considered a SNP. Additional filters can be specified for this step, such as a minimum allele frequency, or sub-setting to only biallelic SNPs (exactly two counts are nonzero). This SNP detection mechanism is hence independent of the input file format, and simply uses the available data. For instance, with sam/bam files, we typically have data at (almost) all loci, and can hence use this to distinguish invariant sites from low quality or missing sites.

### 3.3 Windowing

The data is then assembled into windows along the genome. We implemented different types of windows, depending on the analysis needs, namely, representing (i) intervals of a fixed number of bases, (ii) a fixed number of variants (SNPs) per window, (iii) user-defined regions that can be potentially nested or overlapping, such as genes or LD blocks, (iv) single SNPs, (v) whole chromosomes, and (vi) whole genome. Existing tools only offered one or two of these types of windows. The first three of these types keep data in memory proportional to the window sizes, which is necessary for overlapping windows and for sliding windows to allow a stride between windows smaller than the window size. The remaining window types (single SNPs, whole chromosomes, and whole genome) instead directly stream through the input data, thereby keeping the memory footprint to a minimum. This is a distinguishing feature compared to, e.g. poolfstat, which reads whole files into memory, and hence does not scale to large datasets with many samples (see Supplementary Material).

### 3.4 Statistics computation

Finally, with the data stream processed as described above, the desired statistical estimators are computed. Typically, the statistics are then averaged over the window, in order to obtain per-base-pair estimates. To account for the characteristics of the input data (with missing data; only containing variant loci; etc.), we offer different policies for determining the denominator used for the window averaging: (i) the window size (likely an underestimation), (ii) the number of all available loci in the input, (iii) the number of “valid” high-quality positions (i.e. the number of loci that passed all quality filters; invariants and SNPs), (iv) the number of SNPs only (likely an overestimation), (v) no averaging (i.e. simply report the sum of all per-site values; this allows the user to apply custom averaging later on), and (vi) using a user-defined mask (this allows to set a window-based denominator for instance based on external quality or SNP filters). These policies are covering the most common use cases of data types. When the data has sufficient coverage, we recommend to use (iii), the number of high-quality positions. If specified, this also takes the mask into account, so that SNP-only input data can be properly normalized per window.

## 4 Performance comparison and implementation

**Figure 2.**
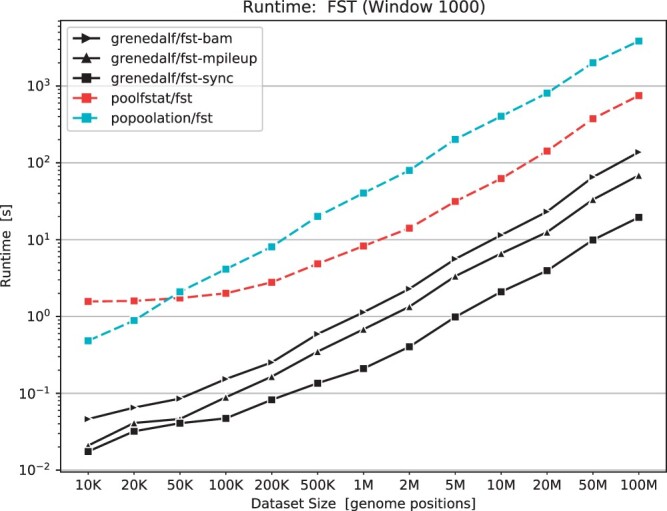
Runtime benchmark (log-log scaled) for computing *F*_ST_ for different input data sizes on real-world data. Here, we compare the single-threaded runtime of grenedalf for computing *F*_ST_ on subsets of two samples from *Arabidopsis thaliana* to existing implementations. With the sync format, grenedalf is about 40x faster than poolfstat, and about 200x faster than PoPoolation. Even with the computationally more demanding pileup and bam formats (which the other two tools do not support as input), grenedalf is significantly faster. The gain over existing tools is even greater for larger datasets, and when using multiple threads. See Supplementary Material for all benchmarks.

In Fig. 2, we compare the runtime of existing tools to grenedalf, which is more than two orders of magnitude faster than previous implementations on real-world data even when run on a single core. More detailed benchmarks are available in the Supplementary Material. Overall, these improvements enable the analysis of datasets much larger, as for instance required in our GrENE-net.org experiment (Czech *et al.* 2022). Furthermore, this will allow for novel types of applications that were previously not feasible, such as running bootstrapping (either over reads, or genomic positions, or both) to obtain confidence intervals for the statistics of interest.

Performance in runtime and memory was one of the major design goals. For instance, the file parsing is highly optimized and executed with asynchronous buffers. All data is read in streams, so that the number of input files, and their sizes, do not significantly affect the amount of required memory. Processor-intensive steps, such as file parsing and the statistics computations, are multi-threaded with a high-performance shared thread pool to leverage modern multi-core systems, and we paid close attention to selecting appropriate data structures for efficiency. Particular care was given to the implementation of the statistics; we optimized computations toward CPU-level parallelism, increased overall numerical stability and range, extended the range of valid inputs for aspects such as the involved binomial computations, and replaced some expensive subroutines by fast closed-form expressions or lookup-tables.

The core implementation of the command line tool grenedalf is part of genesis, our high-performance software library for working with phylogenetic and population genetic data (Czech *et al.* 2020). Written in modern C++, genesis is the best-scoring code across 48 scientific code bases in comprehensive software quality benchmarks (Zapletal *et al.* 2021). A key feature of the underlying software design is its flexibility and modularity. The design allows for further additions of file formats and statistics algorithms without the need to alter any other software component. Therefore, any new addition benefits from the overall architecture and efficiency, and components of the software can be combined as needed. This structure permits users to use the command line tool grenedalf directly on their datasets, but they can also use the functionalities of genesis for their own method development.

## 5 Conclusion and outlook

We presented grenedalf, a command line tool for computing population genetic statistics, which scales to modern pool sequencing datasets, and which provides a plethora of input file formats and convenience options.

In the future, given the ease with which statistics computations can be incorporated into our modular software design, we aim to re-implement more of the existing Pool-seq statistics, such as *f* statistics (Hivert *et al.* 2018, Gautier *et al.* 2022), and implement a Pool-seq-based GWA tool (Schlötterer *et al.* 2014). An under-explored area is the incorporation of short indels, which can potentially be treated as another type of count-based variation. Furthermore, we want to integrate grenedalf with our short-read processing and variant calling pipeline grenepipe (Czech and Exposito-Alonso 2022), which already supports estimating allele frequencies from Pool-seq data via the HAF-pipe tool (Kessner *et al.* 2013, Tilk *et al.* 2019). To this end, it will also be beneficial to develop a proper file format for allele frequencies from Pool-seq, akin to the vcf for individual sequencing.

## Supplementary Material

btae508_Supplementary_Data

## Data Availability

grenedalf is published under the GPL-3, and freely available at github.com/lczech/grenedalf.
