# Supplementary material for: grenedalf: population genetic statistics for the next generation of pool sequencing
Source: Bioinformatics. 2024 Aug 26;40(8):btae508. doi: 10.1093/bioinformatics/btae508 (PMC11357794; doi:10.1093/bioinformatics/btae508)
Supplement: btae508_Supplementary_Data [file btae508_supplementary_data.zip › Pool-Sequencing equations for population genetic statistics; includes assessment of biases of the statistics.pdf]

Pool-Sequencing corrections for population genetic statistics

Lucas Czech[a,b,*], Jeffrey P. Spence[c], and Moisés Expósito-Alonso[a,d,e,f,g,*]

[a]Department of Plant Biology, Carnegie Institution for Science, Stanford, USA.
[b]Section for GeoGenetics, Globe Institute, University of Copenhagen, Denmark.
[c]Department of Genetics, Stanford University, Stanford, USA.
[d]Department of Biology, Stanford University, Stanford, USA.
[e]Department of Global Ecology, Carnegie Institution for Science, Stanford, USA.
[f]Department of Integrative Biology, University of California Berkeley, Berkeley, USA.
[g]Howard Hughes Medical Institute, University of California Berkeley, Berkeley, USA.
[*]To whom correspondence should be addressed: lczech@carnegiescience.edu, and moisesexpositoalonso@gmail.com

Version: 2024-07-23

The latest version of this document can be found at https://github.com/lczech/pool-seq-pop-gen-stats
When citing this document or equations in it, we recommend to also mention the version, as section and equation numbering might change.

Abstract

This document describes our assessment of the specific pool-sequencing (Pool-seq) equations for population genetic measures of diversity (such as θ_π, $\theta_{\text{Watterson}}$, Tajima's D), and differentiation (such as F_{ST}). The aim of these equations is to correct for the noises of pool sequencing, specifically limited sample size (number of individuals pooled, or pool size n) and limited read depth (number c of reads obtained from those individuals that cover a given locus).

We re-render some approaches originally presented and implemented in PoPoolation [14] and PoPoolation2 [15], as well as other publications. We re-derive estimators of genetic diversity, and suggest some improvements that fix standing issues when working with Pool-seq data. Furthermore, we (re-)derive two consistent (asymptotically unbiased) estimators for F_{ST} for pool sequencing data, using two distinct definitions of F_{ST}, that correct for both sources of noise mentioned above. We also evaluate the different estimators for F_{ST} here using simulated data, showing their particular biases. Lastly, we derive novel estimators for F_{ST} that additionally take sequencing error into account.

Our main findings are as follows. (1) Correcting for Pool-seq noise in Tajima's D in order to obtain results that are numerically comparable to values obtained from individual sequencing is difficult, and the approach of PoPoolation has statistical shortcomings. We hence recommend not interpreting their values of Tajima's D quantitatively, and instead compare θ_π and θ_w directly without Pool-seq corrections. (2) Some of the established Pool-seq estimators for F_{ST} are biased upward for small pool sizes and low read depth; we hence recommend using the estimators presented here instead.

The estimators presented here are implemented in our tool grenedalf: https://github.com/lczech/grenedalf

Contents

1 Preliminaries . . . **3**
1.1 Pool Sequencing Data and Notation . . . 3
1.2 Statistical Model of Pool Sequencing . . . 4

2 Pairwise Heterozygosity θ_π . . . **5**

3 Watterson's Theta, $\widehat{\theta}_w$ . . . **8**

4 Tajima's D . . . **9**
4.1 Pool-Sequencing Correction . . . 9
4.2 Integration with Classic Tajima's D . . . 10
4.3 Assumptions and Biases . . . 13
4.4 PoPoolation Bugs . . . 13
4.5 Conclusion and Comprehensive Approach . . . 14

5 Fixation Index F_{ST} for Pool-Seq . . . **15**
5.1 Unbiased estimators of the πs . . . 16
5.2 Final asymptotically unbiased estimators of F_{ST} per SNP and per window . . . 17
5.3 Estimator of F_{ST} as implemented in PoPoolation2 . . . 18
5.4 Asymptotically Unbiased Estimator of F_{ST} by Karlsson *et al.* . . . 20
5.5 Comparison of the Estimators and their Biases . . . 21
5.6 Unbiased estimates of heterozygosity in the presence of sequencing error . . . 26

6 PoPoolation Equations Document . . . **30**

7 References . . . **31**

Note on sources:

- This document is partially based on the PoPoolation equations document `correction_equations.pdf` as found in their code repository; we provide a copy at https://github.com/lczech/popoolation/blob/master/files/correction_equations.pdf. This document derives some of the equations implemented, but also contains equations that are not implemented in PoPoolation but may be of interest for a deeper understanding of the topic.
- Furthermore, we incorporated several implementation details from the reverse-engineered code of PoPoolation and PoPoolation2. We want this document to represent the equations that are actually computed when running these programs (and their re-implementations in grenedalf), as we feel that they need a more thorough assessment than what is available in the current literature.
- Lastly, for the avid reader, apart from these documents and the original publications of PoPoolation [14] and PoPoolation2 [15], we also recommend the npstat [6] and poolfstat [7, 10] publications, which contain further renderings of some of the equations.

1 Preliminaries

1.1 Pool Sequencing Data and Notation

We first define the input that we assume to be given for all subsequent equations. In the software implementation of the equations, these are based on the input data, or set by the user as parameters.

n : Pool size, as given in the dataset (and in the software, provided by the user). This is the number of individuals (haplotypes) that were pooled together for the sequencing of a population. Note on the pool size for different levels of ploidy: The pool sequencing approach assumes the pool size to be the number of distinct haplotypes in the population that have been sampled. Hence, for instance for 100 diploid individuals, the number of haplotypes (and hence the pool size) is 200.

c : Observed read depth: The number of reads sequenced from the pool at a focal position in the genome. This is the main data that we are using. NB: In PoPoolation, npstat, as well as previous versions of this document (up until, and including, version 2023-09-12), this is called "coverage" instead of read depth. This term however is ambiguous and can both mean "coverage depth" (number of reads at each base of the genome) and "coverage breadth" (proportion of the genome that is covered by reads).

b : Minimum allele count, provided by the user. We do not want to consider SNPs with fewer than b alternative reads in the data, as they might be sequencing errors. Note that we assume b to be a user-provided constant, and hence leave it out of (most) function arguments for simplicity.

For most equations we will consider a single pool sequencing sample (one population, or one experiment), but in cases where we consider multiple samples (multiple pool-sequenced populations), we will denote the different samples with subscripts in parentheses, such as $n_{(1)}, n_{(2)}, \dots$ or $c_{(1)}, c_{(2)}, \dots$ to denote different populations or experiments.

Beyond these data-specific and user-specified parameters, we will use the following notation. In general, we will use capital letters for random variables in our model of pool sequencing, which will be described shortly in Section 1.2. We will also differentiate sample quantities (i. e., those that are computed from data) from their population counterparts (i. e., parameters that describe the population from which the data were sampled) with hats. For example, we differentiate the frequency of the A allele in the whole population, f_A, from the empirical frequency of A nucleotides in our pool sequencing sample, $\widehat{f}_A$.

τ : Index over nucleotides, with $\tau \in \{\mathrm{A}, \mathrm{C}, \mathrm{G}, \mathrm{T}\}$ being implicit in any summations.

C_τ : Nucleotide counts, i. e., how many reads have a certain nucleotide τ at a given position in the genome. Note that we generally do not make assumptions about the focal position in the genome being biallelic, unless stated otherwise.

$c = \sum_\tau C_\tau$: The (total) read depth at a given position.

f_τ : Nucleotide frequencies in the population, i. e., the proportion of haplotypes that have nucleotide τ at the focal position.

$\widehat{f}_\tau$: Empirical nucleotide frequencies, i. e., $\widehat{f}_\tau := C_\tau / c$. Naturally, $\sum_\tau \widehat{f}_\tau = 1$.

m : Index of summation over potential levels of read depth c.

k : Index of summation over potential pool sizes n.

We will also make use of the generalized harmonic numbers, which frequently arise in coalescent theory. Define a_1 and a_2 as the sum of (squared) reciprocals of the first n positive integers:

$$a_1(n) := \sum_{k=1}^{m} \frac{1}{k} \tag{1}$$

$$a_2(n) := \sum_{k=1}^{m} \frac{1}{k^2} \tag{2}$$

We use this notation as a compromise between Equation (3.6) of Hahn (2018) [8] and the notation of a_n and b_n used in Achaz (2008) [1] for these quantities.

1.2 Statistical Model of Pool Sequencing

We consider a simple model of pool sequencing as a series of multinomial samplings from a population. In particular we assume the following model, where N_τ is the (unobserved) number of individuals in the pool with the τ allele:

$$(N_A, N_C, N_G, N_T) \sim \text{Multinomial}\left(n, (f_A, f_C, f_G, f_T)\right)$$

$$(C_A, C_C, C_G, C_T)|(N_A, N_C, N_G, N_T) \sim \text{Multinomial}\left(c, \left(\frac{N_A}{n}, \frac{N_C}{n}, \frac{N_G}{n}, \frac{N_T}{n}\right)\right)$$

Throughout, we will repeatedly make use of the following results:

$$\mathbb{E}\left[\widehat{f}_\tau\right] = f_\tau, \tag{3}$$

and

$$\mathbb{E}\left[\left(\frac{c}{c-1}\right)\left(\frac{n}{n-1}\right)\left(1 - \sum_\tau \widehat{f}_\tau^2\right)\right] = 1 - \sum_\tau f_\tau^2. \tag{4}$$

To see Equation 3 we can use the tower property:

$$\mathbb{E}\left[\widehat{f}_\tau\right] = \mathbb{E}\left[\mathbb{E}\left[\widehat{f}_\tau \mid N_\tau\right]\right] = \mathbb{E}\left[\frac{N_\tau}{n}\right] = f_\tau.$$

To obtain Equation 4, we make use of the following fact about binomial distributions. Suppose X is binomially distributed with parameters m and p, then X can be represented as the sum of m independent, identically distributed Bernoulli random variables, $Y_1, \ldots, Y_m$ that take the value 1 with probability p and 0 with probability $1 - p$. We can then see:

$$\begin{aligned}\mathbb{E}\left[X(m-X)\right] &= \mathbb{E}\left[\sum_{i=1}^m \sum_{j=1}^m Y_i(1-Y_j)\right] \\ &= m\mathbb{E}\left[Y_1(1-Y_1)\right] + m(m-1)\mathbb{E}\left[Y_1(1-Y_2)\right]\end{aligned}$$

where the second equality follows from the linearity of expectation and the fact that all of the Y_is are identically distributed. Then, since Y_i is either 0 or 1, $Y_i(1 - Y_i)$ must be zero. Finally, by independence,

$$\mathbb{E}\left[Y_1(1-Y_2)\right] = \mathbb{E}\left[Y_1\right]\left(1 - \mathbb{E}\left[Y_2\right]\right) = p(1-p).$$

Therefore,

$$\mathbb{E}\left[X(m-X)\right] = m(m-1)p(1-p)$$

We can now make use of this result twice along with the tower property to obtain Equation 4:

$$\begin{aligned}
\mathbb{E}\left[\left(\frac{c}{c-1}\right)\left(\frac{n}{n-1}\right)\left(1-\sum_\tau \widehat{f}_\tau^2\right)\right] &= \sum_\tau \left(\frac{c}{c-1}\right)\left(\frac{n}{n-1}\right)\mathbb{E}\left[\widehat{f}_\tau(1-\widehat{f}_\tau)\right] \\
&= \sum_\tau \left(\frac{c}{c-1}\right)\left(\frac{n}{n-1}\right)\left(\frac{1}{c^2}\right)\mathbb{E}\left[C_\tau\left(c-C_\tau\right)\right] \\
&= \sum_\tau \left(\frac{1}{c(c-1)}\right)\left(\frac{n}{n-1}\right)\mathbb{E}\left[\mathbb{E}\left[C_\tau\left(c-C_\tau\right) \mid N_\tau\right]\right] \\
&= \sum_\tau \left(\frac{n}{n-1}\right)\mathbb{E}\left[\frac{N_\tau}{n}\left(1-\frac{N_\tau}{n}\right)\right] \\
&= \sum_\tau \frac{1}{n(n-1)}\mathbb{E}\left[N_\tau\left(n-N_\tau\right)\right] \\
&= \sum_\tau f_\tau(1-f_\tau) = 1-\sum_\tau f_\tau^2.
\end{aligned}$$

The last equality holds as $\sum_\tau f_\tau = 1$.

2 Pairwise Heterozygosity θ_π

First, we derive estimators for the pairwise heterozygosity, θ_π, also called Tajima's π. Formally, pairwise heterozygosity is the probability that two haploids drawn *from the population* have different alleles at a position chosen uniformly at random. We always assume that the population is large so that for a particular site, $\theta_\pi = 1 - \sum_\tau f_\tau^2$, which can be seen by noting that the probability that we pick two individuals with the τ allele is f_τ^2.

If we were to sequence individuals, it would be straightforward to derive unbiased estimators for θ_π, but instead we obtain a pool of reads, which introduces two major issues. The first is that we no longer have access to individuals. If we had sequenced individuals, we could estimate θ_π by looking at two individuals and asking if they have different alleles at a position, but in pool sequencing, we can only look at two different reads and ask if they have different alleles. The issue is that we cannot know whether those reads were from the same individual or from different individuals, so a naive estimator of θ_π that just looks at pairwise differences between reads will be biased because in some fraction of times those reads will have come from the same individual, and hence must have the same allele (ignoring sequencing error). The second major issue is that positions where only a small number of reads have a particular allele could be an artifact of sequencing error. As such, PoPoolation restricts to sites where there are either exactly 0 or at least b reads supporting each allele.

Below we derive PoPoolation's estimator of pairwise heterozygosity, $\widehat{\theta_\pi}$, which addresses both of the issues listed above. Dealing with the bias from pool-sequencing is straightforward. Restricting to positions where there are at least b reads with the minor allele is more complex, however, and requires additional assumptions that will almost certainly not be met in practice. In particular, the $\widehat{\theta_\pi}$ estimator crucially relies on the assumption that the population is evolving neutrally and is at equilibrium – in technical terms, the derivation relies on the sample frequency spectrum being proportional to $1/k$, which is to say that the probability that k out of n individuals have the derived allele at a segregating site has probability proportional to $1/k$. In practice, natural selection, gene flow, or fluctuations in effective population size can all cause deviations from this assumption, and these deviations will result in $\widehat{\theta_\pi}$ being a biased estimator of θ_π.

PoPoolation Pool-seq estimator, $\widehat{\theta_\pi}$

PoPoolation begins by defining

$$\widehat{\theta_\pi}^{\,n\to\infty} := \frac{c}{c-1}\left(1-\sum_\tau \widehat{f}_\tau^2\right), \tag{5}$$

which is equal to the left hand side of Equation 4 up to a factor of $\frac{n}{n-1}$. This indicates that this is a biased estimator of the heterozygosity at this site, but the bias is on the order of $O\left(\frac{1}{n}\right)$, which means that PoPoolation assumes an extremely large pool size, which is why we use the $n \to \infty$ superscript.

At this point, the PoPoolation equations document begins to simplify the above equation, and then breaks it down for biallelic SNPs. However, their (and our) implementation differs from this, and uses the above equation that works with any (not just biallelic) SNPs. We hence do not introduce these simplifications here. Note however that the computation is still only conducted on biallelic sites, as the correction term introduced below assumes this. This means, we are assuming that multiallelic sites are rare, which is true for small mutation rates.

Bias of $\widehat{\theta_\pi}^{\,n\to\infty}$ when restricting to sites with at least b minor allele reads

Other than the bias from the finite pool size, $\widehat{\theta_\pi}^{\,n\to\infty}$ is a reasonable estimator of the pairwise heterozygosity at a given site. To obtain an overall estimate of θ_π, we could average $\widehat{\theta_\pi}^{\,n\to\infty}$ across all sites. PoPoolation seeks to avoid including sites that appear to be segregating solely because of sequencing error, however, which adds a complication. We expect that sequencing errors should be rare, and so it would be extremely unlikely to see b or more reads supporting a particular allele solely due to sequencing error. As such, we can remove all sites with fewer than b reads supporting the minor allele. This is problematic, however, and biases the estimator — while removing such sites certainly removes most or all sites that are segregating solely due to sequencing error, it also removes a large number of sites where the mutation just happens to be at low frequency.

The approach that PoPoolation takes is to assume neutrality and an equilibrium demography (all variation is neutral, and the population is of constant size and in mutation-drift equilibrium), calculate the bias of $\widehat{\theta_\pi}^{\,n\to\infty}$, and introduce a term to correct for that bias. Despite using sequencing error as a motivation, PoPoolation ignores sequencing error in the following derivation. The derivation will assume biallelic sites, and all expectations in this section will be with respect to both the randomness in the Pool-Seq experiment as well as the randomness in the evolutionary process.

To begin, we compute the expectation of $\widehat{\theta_\pi}^{\,n\to\infty}$ when we treat sites with fewer than b reads supporting the minor allele as not contributing to $\widehat{\theta_\pi}^{\,n\to\infty}$. Since we are assuming that we are only looking at biallelic sites, we will use M to denote the number of reads supporting the derived allele. Letting $\mathcal{E}_b$ be the event that at least b reads support the minor allele, we see

$$\mathbb{E}\left[\widehat{\theta_\pi}^{\,n\to\infty}\mathbb{I}\left\{\mathcal{E}_b\right\}\right] = \sum_{m=b}^{c-b}\mathbb{E}\left[\widehat{\theta_\pi}^{\,n\to\infty} \mid M=m\right]\mathbb{P}(M=m) \tag{6}$$

$$= \mathbb{P}\left(\text{Site is a SNP}\right)\sum_{m=b}^{c-b}\mathbb{E}\left[\widehat{\theta_\pi}^{\,n\to\infty} \mid M=m\right]\mathbb{P}\left(M=m \mid \text{Site is a SNP}\right) \tag{7}$$

$$= 2\mathbb{P}\left(\text{Site is a SNP}\right)\sum_{m=b}^{c-b}\frac{m(c-m)}{c(c-1)}\mathbb{P}\left(M=m \mid \text{Site is a SNP}\right) \tag{8}$$

In words, the expected value is computed by summing all possible SNP counts (that exceed the minimum count b) that can occur in a pool with read depth c, weighted by the probability to have each of those counts. As we are using the derived allele count m in the equation above, and either the derived or ancestral allele could be the minor allele, we "sandwich" our potential values for the read depth between b and $c-b$.

The two probabilities used above are computed as follows.

$\mathbb{P}\left(\text{Site is a SNP}\right)$ is the probability of observing a SNP in our pool of n individuals. Here we invoke our neutrality and equilibrium assumptions, where a classical result from coalescent theory gives:

$$\mathbb{P}\left(\text{Site is a SNP}\right) = 1 - \prod_{k=1}^{n-1} \frac{1}{1+\frac{\theta_\pi}{k}} \approx \theta_\pi a_1(n-1) \tag{9}$$

where the final approximation ignores terms on the order of θ_π^2, which implicitly assumes that the mutation rate is small.

$\mathbb{P}\left(M = m \mid \text{ Site is a SNP }\right)$ is the probability of observing m as the derived allele count in a SNP with c reads from a pool of n individuals. We can further break this down by conditioning on the number of individuals in the pool that have the derived allele, which we will call $\widetilde{M}$:

$$\mathbb{P}\left(M = m \mid \text{site is a SNP}\right) = \sum_{k=1}^{n-1} \mathbb{P}\left(M = m \mid \widetilde{M} = k\right) \mathbb{P}\left(\widetilde{M} = k \mid \text{site is a SNP}\right) \tag{10}$$

$$= \frac{1}{a_1(n-1)} \sum_{k=1}^{n-1} \frac{1}{k} \mathbb{P}\left(M = m \mid \widetilde{M} = k\right) \tag{11}$$

where the second line follows from another classic result in coalescent theory that (assuming neutrality, equilibrium, and that θ_π is small) the probability that k individuals in a pool of size n have the derived allele given that a site is s SNP is $\frac{1}{k a_1(n-1)}$.

Finally, $\mathbb{P}\left(M = m \mid \widetilde{M} = k\right)$ is the probability of having m reads support the derived allele when k individuals in the pool have the derived allele. From our binomial sampling model of pool sequencing, this is:

$$\mathbb{P}\left(M = m \mid \widetilde{M} = k\right) = \binom{c}{m} \left(\frac{k}{n}\right)^m \left(\frac{n-k}{n}\right)^{c-m} \tag{12}$$

In words, this follows a binomial distribution, with m successes out of c trials with a success probability of k/n for each trial. That is, we compute how likely it is to observe m counts of the allele in c reads, given a frequency k/n of that allele in the pool. The count of the other allele is implicitly $c - m$.

Starting from Eq. (8), we can now put this together:

$$\mathbb{E}\left[\widehat{\theta_\pi}^{n\to\infty} \mathbb{I}\left\{\mathcal{E}_b\right\}\right] = 2\theta_\pi \sum_{m=b}^{c-b} \frac{m(c-m)}{c(c-1)} \sum_{k=1}^{n-1} \frac{1}{k} \binom{c}{m} \left(\frac{k}{n}\right)^m \left(\frac{n-k}{n}\right)^{c-m}$$

This shows that if we use our estimator $\widehat{\theta_\pi}^{n\to\infty}$ but treat as zero sites where the minor allele is not supported by at least b reads, then the estimate will be biased by a factor of

$$2 \sum_{m=b}^{c-b} \frac{m(c-m)}{c(c-1)} \sum_{k=1}^{n-1} \frac{1}{k} \binom{c}{m} \left(\frac{k}{n}\right)^m \left(\frac{n-k}{n}\right)^{c-m}$$

which we will now use to eliminate said bias.

Unbiased estimate of θ_π when restricting to sites with at least b minor allele reads

Based on the previous section, we can construct an unbiased estimator for θ_π when restricting to sites with at least b reads supporting the minor allele, which we denote by $\widehat{\theta}_{\pi,\text{pool}}$. Let $\mathcal{S}_{bc}$ be the set of sites where the read depth is c, and at least b reads support the minor allele, and let $\widehat{\theta_\pi}^{n\to\infty}(\ell)$ be the above estimator evaluated at the ℓth site (where above we suppressed the

dependence on site because we only considered one site at a time). Finally, let $\mathcal{C}$ denote the set of distinct observed read depth levels across the genome. We then define $\widehat{\theta}_{\pi,\text{pool}}$ as

$$\widehat{\theta}_{\pi,\text{pool}} := \frac{1}{\#\text{sites}} \cdot \sum_{c \in \mathcal{C}} \frac{\sum_{\ell \in \mathcal{S}_{bc}} \widehat{\theta}_{\pi}^{\,n\to\infty}(\ell)}{2 \sum_{m=b}^{c-b} \frac{m(c-m)}{c(c-1)} \sum_{k=1}^{n-1} \frac{1}{k} \binom{c}{m} \left(\frac{k}{n}\right)^{m} \left(\frac{n-k}{n}\right)^{c-m}} \tag{13}$$

The approach to compute this for a window is to sum up all values across the SNPs in the window, and potentially compute an average to obtain a per-base-pair estimate of nucleotide diversity. This is the equation as implemented in PoPoolation as the measure called `pi`, and implemented in our GRENEDALF as well. Note that the denominator only depends on the total read depth c and the pool size n, and hence only needs to be computed once per read depth level, yielding a significant computational speedup.

Window averaging

In order to properly compute the window average to obtain an estimate on the per-base-pair-scale, we divide the sum of the above estimator for all SNPs in the window, and divide by an appropriate denominator. We ideally want to use the number of high-quality positions in the window for this, i.e., all positions that are not missing, passed all potential quality filters, etc. In other words, we want to normalize by the number of positions where we did in fact call a variant, or are certain enough that it is an invariant position. In good data conditions, this is the denominator that is recommended to use.

However, not all input data types might contain the full information that is necessary to calculate this. For instance, formats such as VCF are by their very nature often used to only list the variant positions (SNPs), and so we lack information on the other positions. We would need to know which of these have not been called as variants simply because they are indeed invariant, versus which ones were of insufficient quality to make a proper call. This information can for instance be provided via some external source, such as a mask file. However, in the absence of this information, common strategies in practice are: (1) Simply use the window length itself as the denominator for averaging, which likely produces an underestimation, in particular in low coverage or low quality conditions; (2) Use the number of SNPs instead, which is definitely an overestimate of the per-base-pair diversity (and instead gives a per-SNP estimate), but when interpreted with this in mind, can still be a relevant metric to compare regions to each other.

3 Watterson's Theta, $\widehat{\theta}_w$

Under neutrality and equilibrium demography, θ_π is equal to $4N_e\mu =: \theta$, where θ is four times the effective population size N_e times the per-generation mutation rate μ, and hence, the estimator in the previous section can be used as an estimate of the population-scaled mutation rate. An alternative estimate of the mutation rate is Watterson's theta, $\widehat{\theta}_w$, which is based on the number of segregating sites instead of the pairwise heterozygosity. If we had sequenced n individuals, the usual definition of $\widehat{\theta}_w$ is:

$$\widehat{\theta}_w := \frac{S}{\#\text{ sites} \cdot a_1(n-1)}$$

where S is the number of segregating sites; see e. g., Equation (3.5) of Hahn (2018) [8]. In the pool sequencing case we might hope to just count up the number of sites that have more than one allele to use in place of S, but sequencing error is deeply problematic here. Any place where any read has a sequencing error will look like a segregating site, vastly inflating our estimate of the true number of sites that are segregating in our sample. PoPoolation therefore follows a similar approach as in the case of the pairwise heterozygosity, by only considering a site to be segregating if at least b reads support each of two alleles. Again, this restriction introduces bias, because it will miss many sites where one of the alleles really is at a low frequency — but still segregating — in the pool. As before, we will compute this bias and then correct for it, again assuming neutrality, and equilibrium demography, and that the mutation rate is small enough to only focus on biallelic sites.

Formally, let M_ℓ be the number of reads supporting the derived allele at site ℓ, and define

$$S_b(\ell) := \begin{cases} 1 & \text{if } b \le M_\ell \le c-b \\ 0 & \text{otherwise} \end{cases} \tag{14}$$

We then define our naive Pool-Seq analog of Watterson's θ as

$$\widehat{\theta}_w := \frac{1}{\text{\# sites} \cdot a_1(n-1)} \sum_{\ell} S_b(\ell)$$

Reasoning the same as above, we get the expected value of $\widehat{\theta}_w$ as

$$\mathbb{E}\left[\widehat{\theta}_w\right] = \frac{1}{a_1(n-1)} \cdot \mathbb{P}\left(\text{Site is a SNP}\right) \cdot \sum_{m=b}^{c-b} \mathbb{P}\left(M = m \mid \text{Site is a SNP}\right)$$

with the two probability terms again as in Eq. (9) and Eq. (10), thus leading to

$$= \frac{1}{a_1(n-1)} \cdot \theta \sum_{m=b}^{c-b} \sum_{k=1}^{n-1} \frac{1}{k} \binom{c}{m} \left(\frac{k}{n}\right)^m \left(\frac{n-k}{n}\right)^{c-m} \tag{15}$$

To accommodate different read depth levels throughout the genome, denote the set of different read depth levels as $\mathcal{C}$, and the set of sites with read depth c as $\mathcal{S}_c$. Equation 15 immediately suggests the following unbiased estimator, which we denote $\widehat{\theta}_{w,\text{pool}}$:

$$\widehat{\theta}_{w,\text{pool}} := \frac{1}{\text{\# sites}} \cdot \sum_{c \in \mathcal{C}} \frac{\sum_{\ell \in \mathcal{S}_c} S_b(\ell)}{\sum_{m=b}^{c-b} \sum_{k=1}^{n-1} \frac{1}{k} \binom{c}{m} \left(\frac{k}{n}\right)^m \left(\frac{n-k}{n}\right)^{c-m}} \tag{16}$$

Again, the approach to compute this for a window is to sum up all values across the SNPs in the window, and normalize as explained above. As before, the denominator for the per-site estimate in Eq. (16) only depends on the read depth c, and hence only needs to be computed once per read depth level that is present in the data. This is the equation as implemented in PoPoolation as the measure called `theta`, and implemented in our GRENEDALF as well.

4 Tajima's D

In the following, we describe our assessment of the pool-sequencing corrected equations for Tajima's D as presented in PoPoolation. We however find that their statistical approach has some shortcomings, as described below, and suspect that these significantly change the results. We hence recommend to *not* use the correction terms, but instead use an uncorrected direct comparison of θ_π and θ_w. This will not allow for a quantitative interpretation of the values in terms of the classical (non-pool) Tajima's D, but avoids biases of the statistics. See Section 4.5 for details. We still describe the equations here, for completeness, and elaborate on their issues below.

Above, we have defined pool-sequencing corrected estimators $\widehat{\theta}_{\pi,\text{pool}}$ and $\widehat{\theta}_{w,\text{pool}}$. Now, we want to use them to define a test akin to Tajima's D for pool sequencing. We are here again following the PoPoolation approach, and re-derive their equations.

4.1 Pool-Sequencing Correction

The PoPoolation equations document derives the following estimator. To the best of our knowledge, this is however not implemented in PoPoolation; instead, they compute Tajima's D as presented in the following Section 4.2. We still introduce the approach here, for reference, and in the hope that it might be helpful.

First, we define:

$$\widehat{d}_{\text{pool}} := \widehat{\theta}_{\pi,\text{pool}} - \widehat{\theta}_{w,\text{pool}} \tag{17}$$

and use this to define our statistic:

$$D_{\text{pool}} := \frac{\widehat{d}_{\text{pool}}}{\sqrt{\widehat{\text{Var}}(\widehat{d}_{\text{pool}})}}, \tag{18}$$

with a plug-in estimate of the variance of $\widehat{d}_{\text{pool}}$. To derive this plug-in estimate of the variance, we start with the standard expansion of the variance:

$$\text{Var}(\widehat{d}_{\text{pool}}) = \mathbb{E}\left[\widehat{d}^2_{\text{pool}}\right] - \mathbb{E}\left[\widehat{d}_{\text{pool}}\right]^2$$

At this point, we use that $\mathbb{E}[\widehat{d}_{\text{pool}}] = 0$ (i.e., unbiased) for populations at equilibrium, since both $\widehat{\theta}_{\pi,\text{pool}}$ and $\widehat{\theta}_{w,\text{pool}}$ are unbiased estimates of θ. Therefore, $\mathbb{E}[\widehat{d}_{\text{pool}}]^2 = 0$.

Then, following PoPoolation, we compute the variance as:

$$\begin{aligned}
\text{Var}(\widehat{d}_{\text{pool}}) &= \mathbb{E}(\widehat{d}^2_{\text{pool}}) \\
&= \mathbb{P}\left(\text{Site is a SNP}\right) \sum_{m=b}^{c-b} \mathbb{E}\left[\widehat{d}^2_{\text{pool}} \mid M = m\right] \cdot \mathbb{P}\left(M = m \mid \text{Site is a SNP}\right)
\end{aligned}$$

which can be resolved using equations Eq. (9) and Eq. (10) from previous sections:

$$= \theta \sum_{m=b}^{c-b} \mathbb{E}\left[\left(\widehat{\theta}_{\pi,\text{pool}} - \widehat{\theta}_{w,\text{pool}}\right)^2 \middle| M = m\right] \cdot \sum_{k=1}^{n-1} \frac{1}{k} \binom{c}{m} \left(\frac{k}{n}\right)^m \left(\frac{n-k}{n}\right)^{c-m} \tag{19}$$

Both $\widehat{\theta}_{\pi,\text{pool}}$ and $\widehat{\theta}_{w,\text{pool}}$ are completely determined once we fix M to be m, so the only unknown quantity is θ. PoPoolation suggests using $\widehat{\theta}_{\pi,\text{pool}}$ on the same window on which we are computing D_{pool}, as our estimate of θ to obtain a plug-in estimate of the variance:

$$\widehat{\text{Var}}(\widehat{d}_{\text{pool}}) := \widehat{\theta}_{\pi,\text{pool}} \sum_{m=b}^{c-b} \mathbb{E}\left[\left(\widehat{\theta}_{\pi,\text{pool}} - \widehat{\theta}_{w,\text{pool}}\right)^2 \middle| M = m\right] \cdot \sum_{k=1}^{n-1} \frac{1}{k} \binom{c}{m} \left(\frac{k}{n}\right)^m \left(\frac{n-k}{n}\right)^{c-m}$$

This assumes that all individuals contribute the same number of reads to the pool. As stated above, both $\widehat{\theta}_{\pi,\text{pool}}$ and $\widehat{\theta}_{w,\text{pool}}$ are completely determined once we fix M to be m, so these values can be pre-computed and stored to achieve a computational speedup.

The first summation in Eq. (19) involves computing $\widehat{\theta}_{\pi,\text{pool}}$ and $\widehat{\theta}_{w,\text{pool}}$ repeatedly $c - 2b$ many times, with each of these computations involving the compututation of their respective denominators. However, as c remains constant throughout this computation, these denominators (the correction terms) are shared across positions along the genome with the same read depth and number of derived alleles, so that we can re-use them across positions with identical values of those, to gain a $\approx C$-fold speedup.

At this point, the PoPoolation equation document also introduces an approach to compute Tajima's D based on the above in windows. We here skip this part for brevity.

4.2 Integration with Classic Tajima's D

On large windows, the classic Tajima's D is not a measure of significance (in number of standard deviations away from the null hypothesis), but instead is a measure of the magnitude of the divergence from neutrality. This is because all loci are considered completely linked, even if they are not in reality. However, the above pool-sequencing Tajima's D instead consideres all loci as

completely unlinked. This ignores positive correlations between linked sites, so it gives a different numerical result that has a much higher absolute value compared to classic Tajima's D.

Now, we intend to derive a correction term for the pool-sequence Tajima's D to obtain values that are comparable to classic Tajima's D in non-small windows, that is, we want a measure of the magnitude of the divergence from neutrality. We again follow the PoPoolation approach, and here derive the equations that are actually implemented.

Note that this is the part that we believe to be difficult, and where the presented equations do not achieve the desired goal of producing values that are numerically comparable to classical Tajima's D for individual-based sequencing. The equations are presented here to the best of our understanding, for completeness. In Section 4.5, we then outline why these are not solving the issue, and present some ideas for moving forward.

Approach by Achaz

PoPoolation uses a modified version of the Y^* test of Achaz (2008) [1], which was originally developed as a test for neutrality despite the presence of sequencing errors. This test only works when excluding singletons, that is, we set $b := 2$ for this part.

Following PoPoolation and Achaz (2008) [1], we first define:

$$f^*(n) := \frac{n-3}{a_1(n-1)\cdot(n-1)-n} \tag{20}$$

which is then used to define:

$$\alpha^*(n) := f^{*2}\cdot\left(a_1(n-1)-\frac{n}{n-1}\right)+f^*\cdot\left(a_1(n-1)\cdot\frac{4(n+1)}{(n-1)^2}-2\cdot\frac{n+3}{n-1}\right)-a_1(n-1)\cdot\frac{8(n+1)}{n(n-1)^2}+\frac{n^2+n+60}{3n(n-1)} \tag{21}$$

and:

$$\begin{aligned}\beta^*(n) := {} & f^{*2}\cdot\left(a_2(n-1)-\frac{2n-1}{(n-1)^2}\right)+f^*\cdot\left(a_1(n-1)\cdot\frac{8}{n-1}-a_1(n-1)\cdot\frac{4}{n(n-1)}-\frac{n^3+12n^2-35n+18}{n(n-1)^2}\right)\\ & -a_1(n-1)\cdot\frac{16}{n(n-1)}+a_1(n-1)\cdot\frac{8}{n^2(n-1)}+\frac{2(n^4+110n^2-255n+126)}{9n^2(n-1)^2}\end{aligned} \tag{22}$$

Note that these equations were originally developed for data from individuals, and hence here, n denotes the number of individuals *as if* we were doing individual sequencing.

NB: The PoPoolation document recommends to counter-check the correctness of their equation with the original of Achaz (2008) [1]. In fact, PoPoolation introduced a mistake in the last term of β^*, which we have fixed here. Above is the (hopefully) correct one, following Achaz (2008) [1]. Note that the mistake only concerns the PoPoolation equations document, but not their implementation. However, in their implementation, PoPoolation instead has a slight mistake in the first term of β^*, which should not have a significant effect on the computed value though.

The number of individuals sequenced

The only unresolved parameter is n, which corresponds to the number of individuals sequenced — if we were to do individual sequencing. In our case of pool sequencing, according to PoPoolation, we can simply substitute this with the expected number of distinct individuals sequenced.

To this end, we use the read depth c, as well as the pool size n, which we here use as our substitute for the number of individuals sequenced. Then, we define $\tilde{n}$ as the expected number of individuals from our pool that have been sequenced:

$$\tilde{n} = \sum_{k=1}^{t} \sum_{j=1}^{k} (-1)^{k-j} \cdot k \binom{n}{k} \binom{k}{j} \left(\frac{j}{n}\right)^c \tag{23}$$

where $t = \max(c, n)$; if n is much larger than c, we can assume $\tilde{n} \approx c$.

Computing the expected number of distinct individuals sequenced corresponds to the following statistical question: Given a set of integers $A = \{1, \ldots, n\}$ (corresponding to individuals), pick a set B of C elements from set A with replacement (corresponding to reads); what is the expected number of distinct values (individuals) that have been picked in B (that we have reads from)?

PoPoolation computes this value by brute force using Eq. (23), that is, by trying all possible ways to pick numbers from the set. However, there exists a closed form solution to this question, which yields massive speedups for larger read depths, which we have implemented.

One way to arrive at the closed form expression is as follows: Define an indicator random variable I_i for $1 \leq i \leq n$ as 1 if individual i is present in the set B (that is, if individual i has been sequenced), and as 0 if not. Then, the size of set B is simply $\sum_{i=1}^{n} I_i$.

The probability that I_i equals 1 (that is, that individual i has been sequenced) for any i is given by:

$$P(I_i = 1) = 1 - \left(\frac{n-1}{n}\right)^c \tag{24}$$

In words, this is the complement of *not* picking i in all of the c picks from set A.

The expected size of the set B can then be computed by linearity of expectation for all i, yielding our closed form expression:

$$\tilde{n} = n \left(1 - \left(\frac{n-1}{n}\right)^c\right) \tag{25}$$

This is the equation that we compute in our implementation to arrive at $\tilde{n}$ for a given read depth c and pool size n.

Note that $\tilde{n}$ is bounded by both n and c, meaning that $\forall n \geq 1$ and $\forall c \geq 1$ we have

$$\tilde{n} \leq n \qquad \text{and} \qquad \tilde{n} \leq c \tag{26}$$

The first inequality follows readily from the fact that $0 \leq (\frac{n-1}{n})^c \leq 1$.
The second inequality can be shown using Bernoulli's inequality:

$$\left(\frac{n-1}{n}\right)^c = \left(1 - \frac{1}{n}\right)^c \geq 1 - \frac{c}{n} \tag{27}$$

With this, we get

$$\tilde{n} = n \left(1 - \left(\frac{n-1}{n}\right)^c\right) \leq n \left(1 - \left(1 - \frac{c}{n}\right)\right) = c \tag{28}$$

This also follows from our definition of $\tilde{n}$, the expected number of sequenced individuals. That number cannot be greater than the pool size n itself, and it can also not exceed the number c of reads that we sequenced from that pool.

Final estimator for D

Now that we have a way of computing a reasonable value for the number of individuals sequenced, we can finally define the estimator:

$$\tilde{D}_{\text{pool}} := \frac{\widehat{\theta}_{\pi,\text{pool}} - \widehat{\theta}_{w,\text{pool}}}{\sqrt{|W|^{-1} \cdot \alpha^*(\tilde{n})\widehat{\theta}_{w,\text{pool}} \; + \; \beta^*(\tilde{n})\widehat{\theta}^2_{w,\text{pool}}}} \tag{29}$$

following PoPoolation and Achaz (2008) [1]. This requires $b = 2$; furthermore, PoPoolation suggests using "not too small" windows. We are using the size $|W|$ of the window here, that is, the total length along the window in bases, which is typically much larger than the number of SNPs in the window. As we use a difference in two estimators of Θ in the numerator here, and incorporate the window size in the denominator, we do not need to apply an additional normalization here.

The above is the estimator as implemented in POPOOLATION and also offered in our implementation. However, note that the original implementation has some unresolved issues, as described below in Section 4.4. We furthermore believe that this estimator does not fully achieve its goal of being quantitatively comparable to classical individual-based Tajima's D, as described in Section 4.5. Before describing these more fundamental problems though, we first have a look at the assumptions and biases of the approach as described above.

4.3 Assumptions and Biases

In the above computation of the correction term for Tajima's D for pool sequencing, several assumptions were made that lead to the resulting estimator being conservative, i. e., yielding smaller values than what would be expected from individual sequencing of samples. Based on the explanation in the POPOOLATION equations document (most of the text in this section is adapted from there), we explore the underlying assumptions and biases.

The locally fluctuating read depth is replaced by the minimum read depth. This makes the variance estimator larger, and therefore leads to conservative estimates of Tajima's D.

The random number of different individuals sequenced under a given read depth C is replaced by its expected value $\tilde{n}$. This assumption should not affect the results much: If the pool size is large compared to the read depth, sequencing the same individual more than once is uncommon.

Furthermore the number of different individuals sequenced will have a low variance. As we are working with the minimum read depth, $\tilde{n}$ will be biased downwards, tending to give a conservative estimate of the variance.

At different positions, the subsets from the pool that are sequenced might be different. Their coalescent histories will be correlated but not identical. As the classical equations for Tajima's D are for single samples sharing a common coalescent history, there is less correlation in the data than assumed with the classical formula. This again should make the variance approximation more conservative.

Summing up, the approximate variance in the above equations provides a conservative approximation, and the values for Tajima's D will tend to be smaller than those that would be expected for an experiment based on individual sequencing of single samples.

Lastly, the POPOOLATION code repository contains a plot showing the correlation between the classical Tajima's D and the corrected Tajima's D using the equations described above; please see here, where the x-axis corresponds to the classical value, and the y-axis the the corrected one. This plot has been made with real-world data from Drosophila with a read depth (in their naming: coverage) of 12, a window size of 500 and a minimum count of 1, and corroborates our sense that the POPOOLATION estimator is conservative.

4.4 PoPoolation Bugs

From our assessment of the POPOOLATION code, and from personal communication with Robert Kofler, we found that the implementation of the above $\tilde{D}_{\text{pool}}$ in POPOOLATION $\leq$ v1.2.2 contains several bugs, which significantly alter the numerical results of the computation of Tajima's D. We want to thank Robert for his support regarding our questions, and for quickly fixing the bugs that we have pointed out.

As mentioned above, based on further observed results, we however suspect that there might be other statistical issues in their approach, as described below in Section 4.5. These would need a more careful examination to be verified. At the time being, we have decided to not look further into this, for the reasons described below.

Note: In version 2023-04-12 of this document, we also included a Figure where we examined the effect of the bugs that we had identified until then, by comparing values of Tajima's D with and without these bugs, for different pool sizes, minimum

read depths (back then, unfortunately called “coverage”), and window sizes. We have now decided to remove that figure again, because with the potentially remaining open statistical issues of their approach, the comparison is rendered futile.

As a consequence of these unresolved issues, we recommend not using this Pool-seq corrected estimator of Tajima’s D, see also below. We hence also recommend rerunning any analyses of Tajima’s D that have been conducted with past versions of PoPoolation, particularly if any results rely on the exact numerical values. If necessary, such analyses should be re-evaluated with the insights described here.

4.5 Conclusion and Comprehensive Approach

Above, the goal was to derive an estimator of Tajima’s D for Pool-seq data which is quantitatively comparable to classical Tajima’s D on individual data. The overall difficulty is to find an estimator for the variance that we can use in Eq. (18). In a sense, there is a philosophical question about what Tajima’s D even should be in the pool sequencing case, because the denominator should be the standard deviation (square root of the variance) of the numerator, but in the Pool-seq context, it is unclear what variance we mean. Using the approach by Achaz, and plugging in the “effective number of individuals sequenced”, as suggested by Kofler et al, is a somewhat reasonable idea, albeit not a perfect one.

Firstly, the equations above leave it open what read depth to use for the computation of $\tilde{n}$ in Eq. (25) for computing the Achaz approach. In PoPoolation, they use the minimum allele count b as provided by the user, instead of empirical read depth in the window. We suspect that this leads to a major distortion of the results: As shown in Eq. (26), the value of $\tilde{n}$ is bounded by both the read depth and the pool size; however, a requirement of the approach is that $b := 2$ (see Section 4.2), which hence means that $\tilde{n} \leq 2$, which will rarely reflect the expected number of individuals sequenced.

Hence, empirical alternatives for the read depth likely yield more reasonable results. For instance, in order to use Tajima’s D for outlier detection, one typically wants to be conservative. In that case, we should assume loci to be as correlated as possible, and hence use the smallest read depth in the given window instead, and plug that into the Achaz approach.

Furthermore, if we ignore all of the noise from pool sequencing, then the denominator should just be based on the equations by Achaz as explained above, but using the pool size n instead of the effective pool size $\tilde{n}$. This probably works well enough if read depth is high, as the noise induced by pool sequencing is relatively small compared to the noise introduced by demography (see below). If however we want to include the noise from pool sequencing, which in particular is relevant at low read depths, then it becomes quite complicated, and the approach as described above starts to break down.

For a comprehensive estimator of the variance, we would have to know about the covariance of frequencies across sites. In the Pool-seq context, this has two components: (1) The first is a demographic component — how has the randomness of the populations affected these two sites? The approach by Achaz answers that question (in isolation). (2) The second is a Pool-seq component — how does the randomness in the allele frequencies at these two sites covary?

These components are tricky to reconcile: By sequencing some subset of individuals at one site, and a different subset of individuals at another site, the estimated frequencies will covary in a different way than if the “same” subset of individuals were sequenced at both sites. We would hence need to somehow integrate over the number of overlapping individuals for every pair of sites, which is not feasible with Pool-seq data.

Hence, finding a comprehensive solution that *actually* yields the intended quantitatively comparable values of Tajima’s D might involve knowledge of population history and simulations under a neutral model. We hence do not further pursue this approach at the time being.

In conclusion, we suggest the following:

- The approach of PoPoolation to use the minimum read depth b to compute $\tilde{n}$ is likely not reasonable.
- Instead, using the pool size to replace the whole term, i. e., $\tilde{n} = n$, is better under high read depths. This basically assumes that the number of individuals sequenced is equal to the pool size.
- Alternatively, for a more conservative approach, we can compute $\tilde{n}$ as in Eq. (25), by using pool size n, but set c to the minimum empirical read depth found in the given window.

- By far the easiest and safest option in our opinion is to (for now) abandon the goal of quantitative comparability with the classic Tajima's D, and instead directly use the difference of $\theta_{\pi,\text{pool}}$ and $\theta_{w,\text{pool}}$. As long as read depth is relatively equal along the regions being compared, this should enable the detection of outliers and other features of interest.

Lastly, we want to invite the research community to further scrutinize these approaches, and provide suggestions for how to move forward, in order to get a Pool-seq equivalent of Tajima's D that is quantitatively comparable to the classic estimator.

5 Fixation Index F_{ST} for Pool-Seq

In this section, we will derive unbiased estimators of various measures of heterozygosity in two populations for Pool-sequencing data. These will then be combined to obtain "sample-size" and "pool-size" corrected estimators of two definitions of F_{ST}. On top of these two estimators for F_{ST} in the pool-sequencing context, we also walk through the two existing estimators as suggested by Kofler *et al.* (2011b) [15] and Karlsson *et al.* (2007) [12]. Both are implemented in PoPoolation2, and are called the "classical" or "conventional pool sequencing" approach, and the "Karlsson approach adapted to digital data", respectively, in Kofler *et al.* (2011b) [15]. We compare all four approaches to each other, and show that the "classical" approach is biased for lower read depths or small pool sizes, and the Karlsson approach is biased for small pool sizes (bias on the order of 1/pool size). See also Hivert *et al.* (2018) [10] for an assessment of F_{ST} in the pool-sequencing context.

There are several non-equivalent *definitions* of F_{ST}. The overall goal is to measure some degree of differentiation between two populations, which can be represented as a proportion of variation that cannot be explained by variation within populations. What is unclear is a proportion of *what* variation? There are two natural candidates leading to two related, but distinct definitions of F_{ST}. The first definition, which we will call $F_{\text{ST}}^{\text{Nei}}$ following Nei (1973) [16], considers the proportion of the total variation in the two populations. This statistic is also called G_{ST}, see for example Equation (5.5) of Hahn (2018) [8]. The second definition, which we will call $F_{\text{ST}}^{\text{Hudson}}$ following Hudson *et al.* (1992) [11], considers the proportion of the variation between populations, see also Cockerham (1969) [5] and Weir and Hill (2002) [17]. This second definition is also considered in Karlsson *et al.* (2007) [12], which we examine below in Section 5.4.

To make this more formal, we can consider the probability that two haploids carry different alleles. We could consider drawing the two haploids from the same population (with the population chosen at random), which we call π_{within}; or we could consider drawing the two haploids from *different* populations, which we call π_{between}; or finally we could consider drawing the two haploids totally at random from either population (potentially the same populations, potentially different populations) which we call π_{total}. See Bhatia *et al.* (2013) [3] for more background information on this.

Our two definitions of F_{ST} are then

$$F_{\text{ST}}^{\text{Nei}} := 1 - \frac{\pi_{\text{within}}}{\pi_{\text{total}}} \tag{30}$$

$$F_{\text{ST}}^{\text{Hudson}} := 1 - \frac{\pi_{\text{within}}}{\pi_{\text{between}}} \tag{31}$$

If we consider a single locus with up 4 alleles, with frequencies $f_{\tau(p)}$ (possibly zero) with τ denoting the allele with $\tau \in \{A, C, G, T\}$ and p denoting the population with subscripts 1 and 2, we can calculate the various πs as follows

$$\pi_{\text{within}} = \frac{1}{2}\left[\left(1 - \sum_{\tau} f_{\tau(1)}^2\right) + \left(1 - \sum_{\tau} f_{\tau(2)}^2\right)\right] \tag{32}$$

$$\pi_{\text{between}} = 1 - \sum_{\tau} f_{\tau(1)} f_{\tau(2)} \tag{33}$$

$$\pi_{\text{total}} = \frac{1}{2}\pi_{\text{within}} + \frac{1}{2}\pi_{\text{between}} \tag{34}$$

which are then used in our above definitions of F_{ST}.

5.1 Unbiased estimators of the πs

Since both definitions of F_{ST} rely on these πs, we will need to derive unbiased estimates for them. We will show below that the following are unbiased estimators of the corresponding quantities without hats:

$$\begin{aligned}\widehat{\pi}_{\text{within}} := \frac{1}{2}\Bigg[&\left(\frac{n_{(1)}}{n_{(1)}-1}\right)\left(\frac{c_{(1)}}{c_{(1)}-1}\right)\left(1-\sum_{\tau}\widehat{f}^{2}_{\tau(1)}\right) \\ + \quad &\left(\frac{n_{(2)}}{n_{(2)}-1}\right)\left(\frac{c_{(2)}}{c_{(2)}-1}\right)\left(1-\sum_{\tau}\widehat{f}^{2}_{\tau(2)}\right)\Bigg] \end{aligned} \tag{35}$$

$$\widehat{\pi}_{\text{between}} := 1 - \sum_{\tau}\widehat{f}_{\tau(1)}\widehat{f}_{\tau(2)} \tag{36}$$

$$\widehat{\pi}_{\text{total}} := \frac{1}{2}\widehat{\pi}_{\text{within}} + \frac{1}{2}\widehat{\pi}_{\text{between}} \tag{37}$$

In the following, we derive these estimators.

Unbiased estimator of $\widehat{\pi}_{\text{within}}$

We have derived previously that

$$\mathbb{E}\left[\left(\frac{n_{(1)}}{n_{(1)}-1}\right)\left(\frac{c_{(1)}}{c_{(1)}-1}\right)\left(1-\sum_{\tau}\widehat{f}^{2}_{\tau(1)}\right)\right] = \left(1-\sum_{\tau}f^{2}_{\tau(1)}\right)$$

within a single population. It follows immediately that averaging these estimators across the two populations is unbiased for π_{within}.

Unbiased estimator of $\widehat{\pi}_{\text{between}}$

Since the two pools are independent, we have that

$$\mathbb{E}\left[\widehat{\pi}_{\text{between}}\right] = 1 - \sum_{\tau}\mathbb{E}\left[\widehat{f}_{\tau(1)}\right]\mathbb{E}\left[\widehat{f}_{\tau(2)}\right]$$

The frequency of alleles within a pool is an unbiased estimate for the frequency in the population, so

$$\mathbb{E}\left[\widehat{f}_{\tau(p)}\right] = f_{\tau(p)}$$

showing that $\widehat{\pi}_{\text{between}}$ is unbiased for π_{between}.

Unbiased estimator of $\widehat{\pi}_{\text{total}}$

That $\widehat{\pi}_{\text{total}}$ is unbiased for π_{total} follows immediately from the definition of π_{total} in Eq. (34) and the unbiasedness of $\widehat{\pi}_{\text{within}}$ and $\widehat{\pi}_{\text{between}}$.

Intuition and Reasoning

In the above equations, for each of the two populations, we apply a double Bessel's correction for our estimate of $\widehat{\pi}_{\text{within}}$ in Eq. (35), to correct for the elevated variance that comes from the double sampling process of picking individuals from a population, and of picking reads from those individuals (during sequencing). Because in both steps, we pick finite numbers, resulting in higher variance in the estimates, which hence needs correcting.

However, the estimate of $\widehat{\pi}_{\text{between}}$ in Eq. (36) does not have those corrections. One way to gain some intuition about why the Bessle's corrections are not needed in that case is as follows.

One way to think about π in a single population is as the probability to get different alleles when picking two random haploids from the population. In a large population, it does not matter whether we pick those two haploids with replacement or without replacement: in that case, even if we pick the two haploids with replacement, it is unlikely that we would happen to pick the same haploid twice. In that sense, π is measuring the probability that two different haploids have different alleles.

Now, to estimate the population-level π_{within}, we could look at a smaller sample of haploids, and calculate the probability that two randomly chosen haploids have different alleles. However, if our sample size is small, then the difference between sampling with or without replacement becomes relevant, because there is a $1/n$ chance that we pick the same haploid twice if we are sampling with replacement. If we pick the same haploid twice, they obviously cannot have different alleles. This means that if we compute our probabilities by sampling with replacement, then we underestimate by a factor of $1 - 1/n = \frac{n-1}{n}$ because in $1/n$ of our comparisons we are actually comparing a haplotype to itself. As a result, we would need to multiply our estimate by $\frac{n}{n-1}$ to undo this underestimation. Something similar happens again when we move to pool sequencing, where our equations are (implicitly) in terms of sampling with replacement, but now we are sampling reads, and so we end up (by the same argument) with needing to multiply by a factor of $\frac{c}{c-1}$.

This however is different in the case of π_{between}. In that case, we are comparing a haploid from one population to a haploid from a different population. Hence, we are comparing a single haploid (or single read) from one population to a single haploid (or read) from another population. If we only draw a single sample, then there is no difference between sampling with or without replacement, and so our equations (which again are implicitly based on sampling with replacement) do not need to be corrected to match the idea of sampling without replacement.

In summary, when considering two entities at a time from the same pool/population, we need to include Bessel's corrections such as for pool size and read depth. However, if we are only considering one entity at a time from each pool/population, then we do not need those corrections.

5.2 Final asymptotically unbiased estimators of F_{ST} per SNP and per window

These estimators then immediately suggest the following ratio estimators for the different definitions of F_{ST}:

$$\widehat{F}_{\text{ST}}^{\text{Nei}} := 1 - \frac{\widehat{\pi}_{\text{within}}}{\widehat{\pi}_{\text{total}}} \tag{38}$$

$$\widehat{F}_{\text{ST}}^{\text{Hudson}} := 1 - \frac{\widehat{\pi}_{\text{within}}}{\widehat{\pi}_{\text{between}}} \tag{39}$$

All of this has been for a single site, but we are often interested in combining information across SNPs within a window W (or possibly genome wide). In such a case, define $\widehat{\pi}_{\text{within}}^{\ell}$ to be $\widehat{\pi}_{\text{within}}$ as above but for SNP $\ell \in W$. Define $\widehat{\pi}_{\text{between}}^{\ell}$ and $\widehat{\pi}_{\text{total}}^{\ell}$ analogously. We then combine information across the SNPs in the window W as

$$\widehat{F}_{\text{ST}}^{\text{Nei}} = 1 - \frac{\sum_{\ell \in W} \widehat{\pi}^{\ell}_{\text{within}}}{\sum_{\ell \in W} \widehat{\pi}^{\ell}_{\text{total}}} \tag{40}$$

$$\widehat{F}_{\text{ST}}^{\text{Hudson}} = 1 - \frac{\sum_{\ell \in W} \widehat{\pi}^{\ell}_{\text{within}}}{\sum_{\ell \in W} \widehat{\pi}^{\ell}_{\text{between}}} \tag{41}$$

See Bhatia *et al.* (2013) [3] for a practical and theoretical justification for using this "ratio of averages" instead of using an "average of ratios". These are our asymptotically unbiased estimators for F_{ST} for Pool-seq data, which take the finite sampling of individuals from the population, and the finite sampling of reads from each individual in the pool, into account.

Furthermore, as explained above in Section 2, when computing values per window, we here too need to take missing and low quality data into account when working with real-world data. To this end, we again want to normalize the involved values of π using only the high-quality positions, that is, the variant and invariant positions that passed all quality filters. However, we are dealing with an estimator here that operators on two pools simultaneously. Hence, the window normalization needs to be applied individually per pool in Eq. (35), by using the number of positions in each population to normalize the two components of the equation separately from each other. Then, in Eq. (36), we instead normalize using the number of positions that passed the quality filters in *both* populations.

In the following, we describe our assessment of the estimators previously implemented in POPOOLATION2. Note that these two estimators do not use the window normalization as explained here, as they do not correct for the read depths per pool individually either. They hence implicitly assume that the window normalization would cancel out anyway, and do not apply it at all. This makes our estimators as described above better suited for data with low coverage or low quality.

5.3 Estimator of F_{ST} as implemented in PoPoolation2

The implementation in POPOOLATION2 [15] offers two ways to estimate F_{ST}: What they call the "classical" or "conventional" approach by Hartl and Clark (2007) [9], and an approach adapted to digital data following Karlsson *et al.* (2007) [12]. In this and the next section, we discuss these estimators. We later show that they are biased, and hence recommend using the estimates as introduced above instead. For comparability and historical backwards compatibility, we however still offer both these estimators in our implementation in GRENEDALF.

First, we present the "classical" approach as implemented in POPOOLATION2, labelled with superscript "Kofler" here, following Kofler *et al.* (2011b) [15]. We compute F_{ST} for two subpopulations, which we here again denote with subscripts (1) and (2), and the total population with (T). We expect pool sizes $n >= 2$.

For each SNP in a given window, POPOOLATION2 computes:

$$\widehat{\pi}_{(1)}^{\text{Kofler}} := \frac{c_{(1)}}{c_{(1)} - 1} \cdot \left(1 - \sum_{\tau} \widehat{f}_{\tau(1)}^2\right) \tag{42}$$

$$\widehat{\pi}_{(2)}^{\text{Kofler}} := \frac{c_{(2)}}{c_{(2)} - 1} \cdot \left(1 - \sum_{\tau} \widehat{f}_{\tau(2)}^2\right) \tag{43}$$

$$\widehat{\pi}_{(T)}^{\text{Kofler}} := \frac{c_{(T)}}{c_{(T)} - 1} \cdot \left(1 - \sum_{\tau} \widehat{f}_{\tau(T)}^2\right) \tag{44}$$

with

$$c_{(T)} := \min\left(c_{(1)}, c_{(2)}\right)$$

$$\widehat{f}_{\tau(T)} := \frac{1}{2}\left(\widehat{f}_{\tau(1)} + \widehat{f}_{\tau(2)}\right)$$

These quantities are accumulated over the window W, with (ℓ) denoting the above at SNP ℓ

$$\widehat{\pi}_{W(1)}^{\text{Kofler}} = \frac{n_{(1)}}{n_{(1)} - 1} \cdot \sum_{\ell \in W} \widehat{\pi}_{(1)}^{\text{Kofler}}(\ell) \tag{45}$$

$$\widehat{\pi}_{W(2)}^{\text{Kofler}} = \frac{n_{(2)}}{n_{(2)} - 1} \cdot \sum_{\ell \in W} \widehat{\pi}_{(2)}^{\text{Kofler}}(\ell) \tag{46}$$

$$\widehat{\pi}_{W(T)}^{\text{Kofler}} = \frac{n_{(T)}}{n_{(T)} - 1} \cdot \sum_{\ell \in W} \widehat{\pi}_{(T)}^{\text{Kofler}}(\ell) \tag{47}$$

with

$$n_{(T)} = \min\left(n_{(1)}, n_{(2)}\right)$$

Finally, the estimate of $F_{\text{ST}}$is computed as:

$$\widehat{F}_{\text{ST}}^{\text{Kofler}} = \frac{\widehat{\pi}_{W(T)}^{\text{Kofler}} - \frac{1}{2}\left(\widehat{\pi}_{W(1)}^{\text{Kofler}} + \widehat{\pi}_{W(2)}^{\text{Kofler}}\right)}{\widehat{\pi}_{W(T)}^{\text{Kofler}}} \tag{48}$$

Note that following the notation of the previous section, $\frac{1}{2}\left(\widehat{\pi}_{W(1)}^{\text{Kofler}} + \widehat{\pi}_{W(2)}^{\text{Kofler}}\right)$ is identical to the windowed version of $\widehat{\pi}_{\text{within}}$ and hence is unbiased. Unfortunately, $\widehat{\pi}_{W(T)}^{\text{Kofler}}$ however is not an unbiased estimator of π_{total}. There are two main issues. First, recall that $\pi_{\text{total}} = \frac{1}{2}\left(\pi_{\text{between}} + \pi_{\text{within}}\right)$. The naive estimator of π_{between} turns out to be unbiased as shown in the previous section, but yet in the PoPoolation estimator, there is a Bessel correction acting on the whole estimator — this effectively biases the contribution of the part estimating π_{between} upward. Second, π_{within} has two components corresponding to the two populations. Since these have different read depths and different sample sizes, the components estimating each of these have different biases that need to be corrected separately. Instead, PoPoolation uses the minimum read depth and the minimum sample size for both. This again will result in an upward bias for the sample with higher read depth and/or larger pool size. Overall, these two errors can result in substantial upward bias for $\widehat{\pi}_{W(T)}^{\text{Kofler}}$, which will result in substantial upward bias for $\widehat{F}_{\text{ST}}^{\text{Kofler}}$, as we show later. This was also pointed out by Hivert *et al.* (2018) [10]. It is therefore recommended to use the estimator presented in the previous section instead.

5.4 Asymptotically Unbiased Estimator of F_{ST} by Karlsson *et al.*

Another estimator for F_{ST} that is offered in POPOOLATION2 is based on the equations used in Karlsson *et al.* (2007) [12], see the last page of the Supplemental Information of Karlsson *et al.* for their derivation. We here briefly also go through the derivation.

We here call this estimator using the superscript "Karlsson", which is again defined for two subpopulations denoted with subscripts (1) and (2). We are here only looking at biallelic SNPs. Instead of τ for the four nucleotides, we hence use q for the frequency of the major (reference) allele and p for the frequency of the minor (alternative) allele. We will also use $\widehat{q}$ and $\widehat{p}$ as the corresponding empirical estimates of q and p (i. e., the number of reads supporting the major or minor allele divided by the read depth).

We start with the definition of $F_{\text{ST}}^{\text{Karlsson}}$ from Karlsson *et al.* for the SNPs in a window W:

$$F_{\text{ST}}^{\text{Karlsson}} = \frac{\sum_W N_k}{\sum_W D_k} \tag{49}$$

where the the numerator N_k and denominator D_k for a single site k in W are:

$$N_k = p_{(1)} \cdot \left(q_{(2)} - q_{(1)}\right) \; + \; p_{(2)} \cdot \left(q_{(1)} - q_{(2)}\right) \tag{50}$$

$$\begin{aligned} D_k &= p_{(1)} q_{(2)} + q_{(1)} p_{(2)} \\ &= N_k + p_{(1)} q_{(1)} + p_{(2)} q_{(2)}. \end{aligned} \tag{51}$$

It is not obvious, but follows from simple algebra that this definition is equivalent to $F_{\text{ST}}^{\text{Hudson}}$ defined above.

These are estimated as follows, using the numerator $\hat{N}_k$ and denominator $\hat{D}_k$ at a single site:

$$\widehat{N}_k = \left(\widehat{q}_{(1)} - \widehat{q}_{(2)}\right)^2 - \left(\frac{h_{(1)}}{c_{(1)}} + \frac{h_{(2)}}{c_{(2)}}\right) \tag{52}$$

$$\widehat{D}_k = \widehat{N}_k + h_{(1)} + h_{(2)} \tag{53}$$

with two additional helpers:

$$h_{(1)} = \frac{c_{(1)}}{\left(c_{(1)} - 1\right)} \widehat{q}_{(1)} \widehat{p}_{(1)}$$

$$h_{(2)} = \frac{c_{(2)}}{\left(c_{(2)} - 1\right)} \widehat{q}_{(2)} \widehat{p}_{(2)}$$

And finally, these are used to define estimator $\widehat{F}_{\text{ST}}^{\text{Karlsson}}$ for a window W:

$$\widehat{F}_{\text{ST}}^{\text{Karlsson}} = \frac{\sum_W \widehat{N}_k}{\sum_W \widehat{D}_k} \tag{54}$$

According to Karlsson *et al.*, when the read depths $c_{(1)}$ and $c_{(2)}$ (called "sample sizes" there) are equal, the estimator reduces to the estimator of F_{ST} given by Weir and Hill (2002) [17]. Karlsson *et al.* further state that by the Lehmann-Scheffé theorem [4,

Theorem 4.2.2], it follows that $\widehat{N}_k$ and $\widehat{D}_k$ are uniformly minimum variance unbiased estimators of N_k and D_k, respectively, and hence conclude that their estimator $\hat{\mathrm{F}}_{\mathrm{ST,K}}$ is also asymptotically unbiased.

This estimator is very similar (after some algebra) to our $\widehat{F}_{\mathrm{ST}}^{\mathrm{Hudson}}$. It however assumes the pool size to be infinite, that is, it is missing the corrections for pool size.

5.5 Comparison of the Estimators and their Biases

As mentioned above, both the Kofler estimator in PoPoolation2 and the Karlsson estimator have biases. We here explore their effects via simulations, and further show that our estimators are approximately unbiased under the Pool-seq assumptions so long as sequencing error rates are low.

Simulation Setup

To test the accuracy of the different estimators of F_{ST} we performed two sets of simulations. In both sets of simulations we assumed a biallelic mutation model, and a simple model of sequencing. Our sequencing model assumes that (1) each position is independent, (2) the number of reads at a given position is Poisson distributed with the mean of that distribution being the "Read Depth" of that simulation, (3) each read is equally likely to come from any individual in the pool, and (4) with probability "Seq Error" a read shows the opposite allele as the individual it was sampled from, but otherwise matches the individual's allele, and this error process is independent across reads. Across both sets of simulations, we vary the sequencing error in $\left\{0, 10^{-5}, 10^{-4}, 10^{-3}\right\}$, the number of haploid individuals in the pool in $\{10, 100, 1000\}$, and the mean sequencing depth in $\{10, 100, 1000\}$. In both sets of simulations, we obtain population-level allele frequencies at each site, and then compute the true values of either $F_{\mathrm{ST}}^{\mathrm{Nei}}$ or $F_{\mathrm{ST}}^{\mathrm{Hudson}}$ using eqs. (30) to (34). We here use "0" and "1" to denote the major and minor allele, respectively.

In the first set of simulations, which we call the "simple" simulations, we simulate 1000 sites in two populations. Let $f_j^{(p)}$ be the frequency of one of the "1" allele at position j in population p. To obtain correlated allele frequencies in the two populations we simulated the frequencies as

$$f_j^{(1)} \overset{i.i.d.}{\sim} \mathrm{Beta}(0.1, 0.5)$$

$$f_j^{(2)} | f_j^{(1)} \sim \mathrm{Beta}\left(\frac{\gamma f_j^{(1)}}{1 - f_j^{(1)} + 10^{-10}}, \gamma\right),$$

where γ controls the degree of correlation between the frequencies in the two populations. As γ increases, the correlation between the frequencies decreases. This parameterization is chosen so that $\mathbb{E}\left[f_j^{(2)} \mid f_j^{(1)}\right] = f_j^{(1)}$ up to the error introduced by the 10^{-10} which is included for numerical stability. Then, to obtain the frequency of the "1" allele in our pool, we performed independent binomial sampling at each site for each population. Finally, we obtained the number of reads with the "0" and "1" alleles as described above. We varied γ in $\{0.1, 0.325, 0.55, 0.775, 1\}$, and performed 10 simulations each for each setting of γ, sequencing error, pool size, and sequencing depth, resulting in 1800 simulations.

In the second set of simulations we used `msprime` [2, 13] to simulate data under a population genetic model. In this model, we simulate two populations of constant size that diverged at a time, t_{div}, in the past, with the ancestral population having the same, constant size. Across simulations, we varied t_{div} in $\{0, 0.25, 0.5, 0.75, 1.0\}$. We used a population-scaled mutation rate, θ, of 0.001, a population-scaled recombination rate, ρ, of 0.0001, and simulated sequences of length 1 Mb. Since `msprime` is a coalescent simulator, it samples individual-level data. As such, for a given simulation we directly obtain the alleles of the individuals at each of the 10^6 sites for the individuals that make up our pool sequencing sample. We then draw reads for each of those sites as described above. To obtain population-level allele frequencies we simulate an additional 1000 haploid individuals in each population beyond those that make up the pool, and we use the empirical frequencies of the alleles in this 1000 + pool size sample as the "true" allele frequencies in each population.

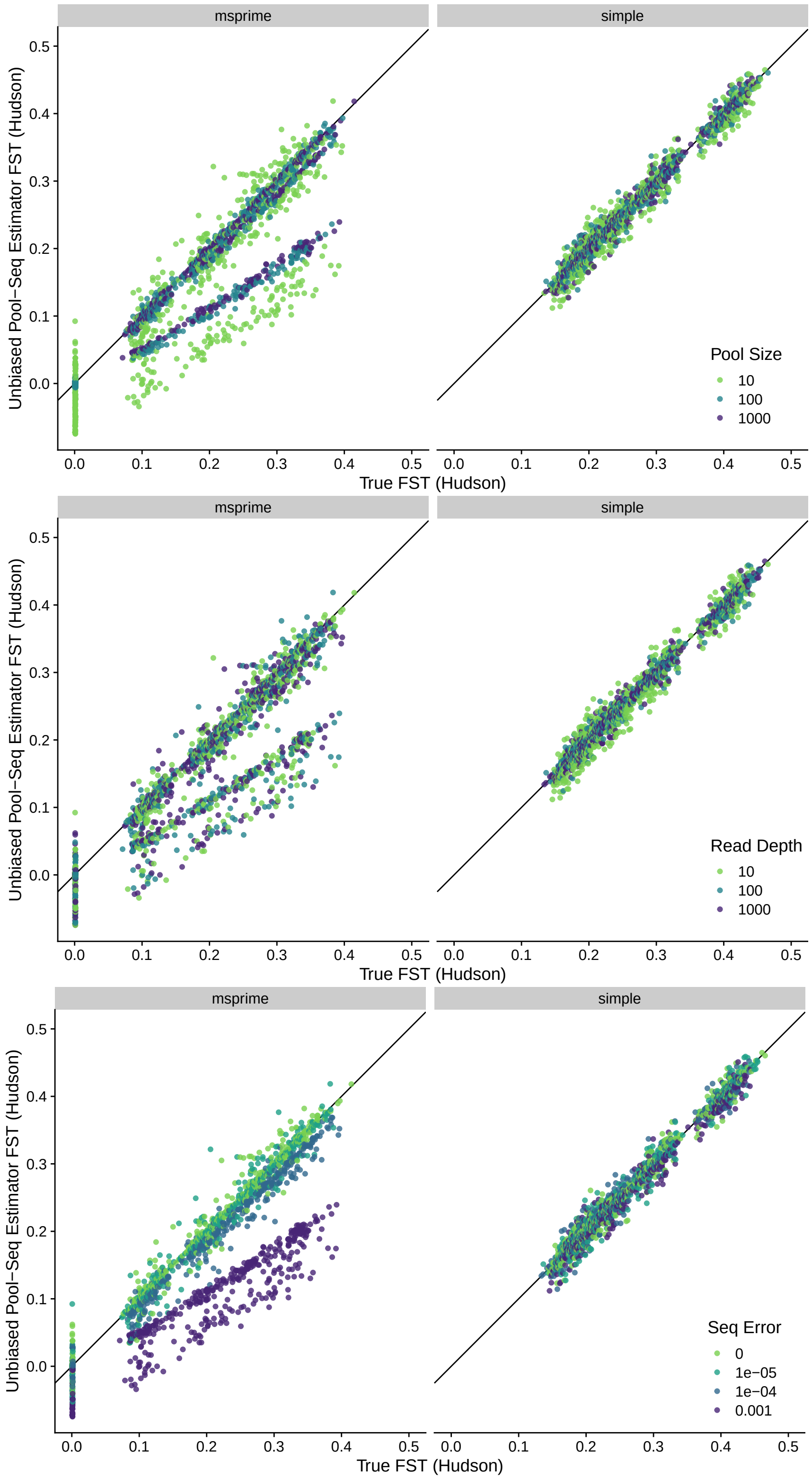

Figure 1: Unbiased Pool-Seq Estimator for FST (Hudson) vs true FST (Hudson).

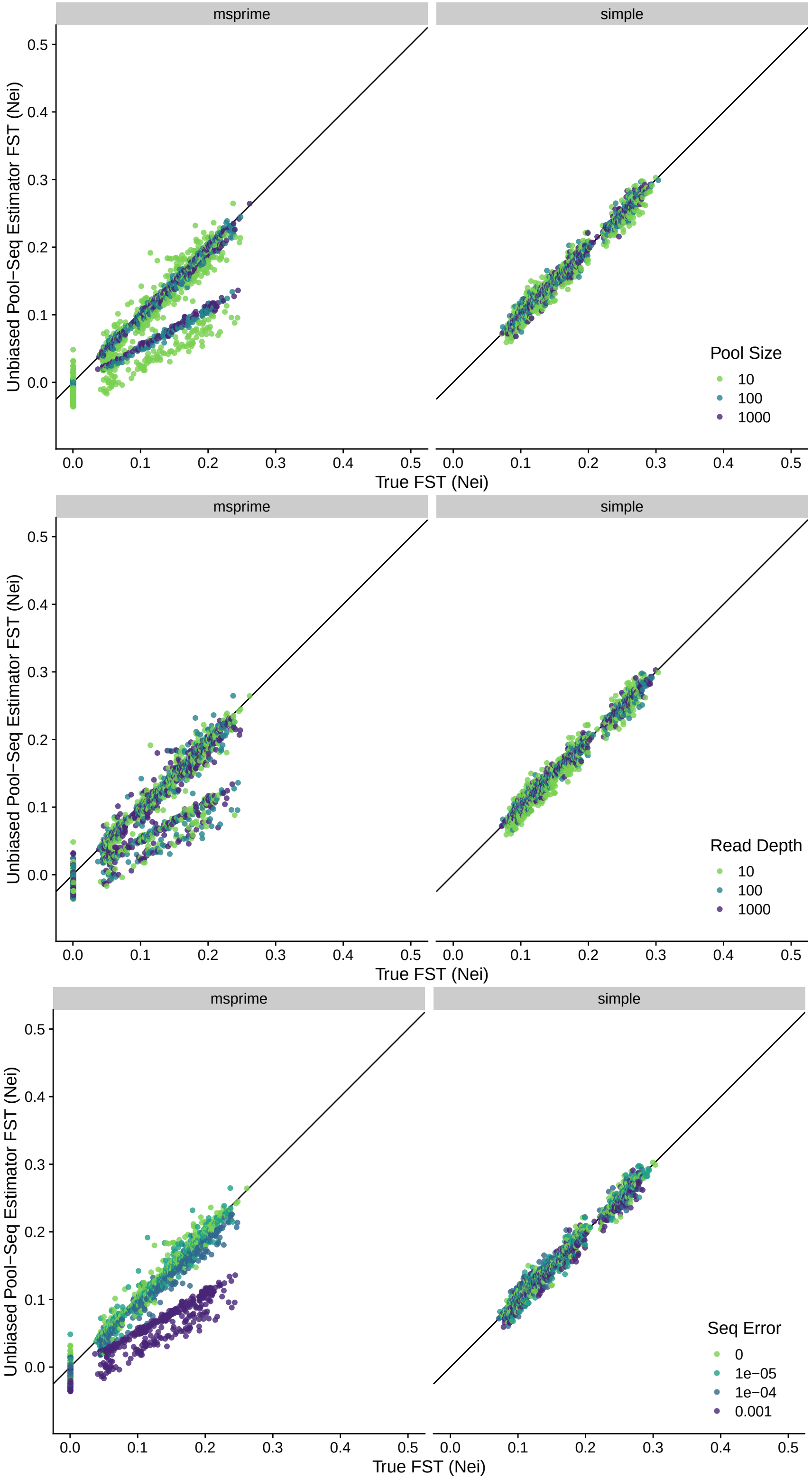

Figure 2: Unbiased Pool-Seq Estimator for FST (Nei) vs true FST (Nei).

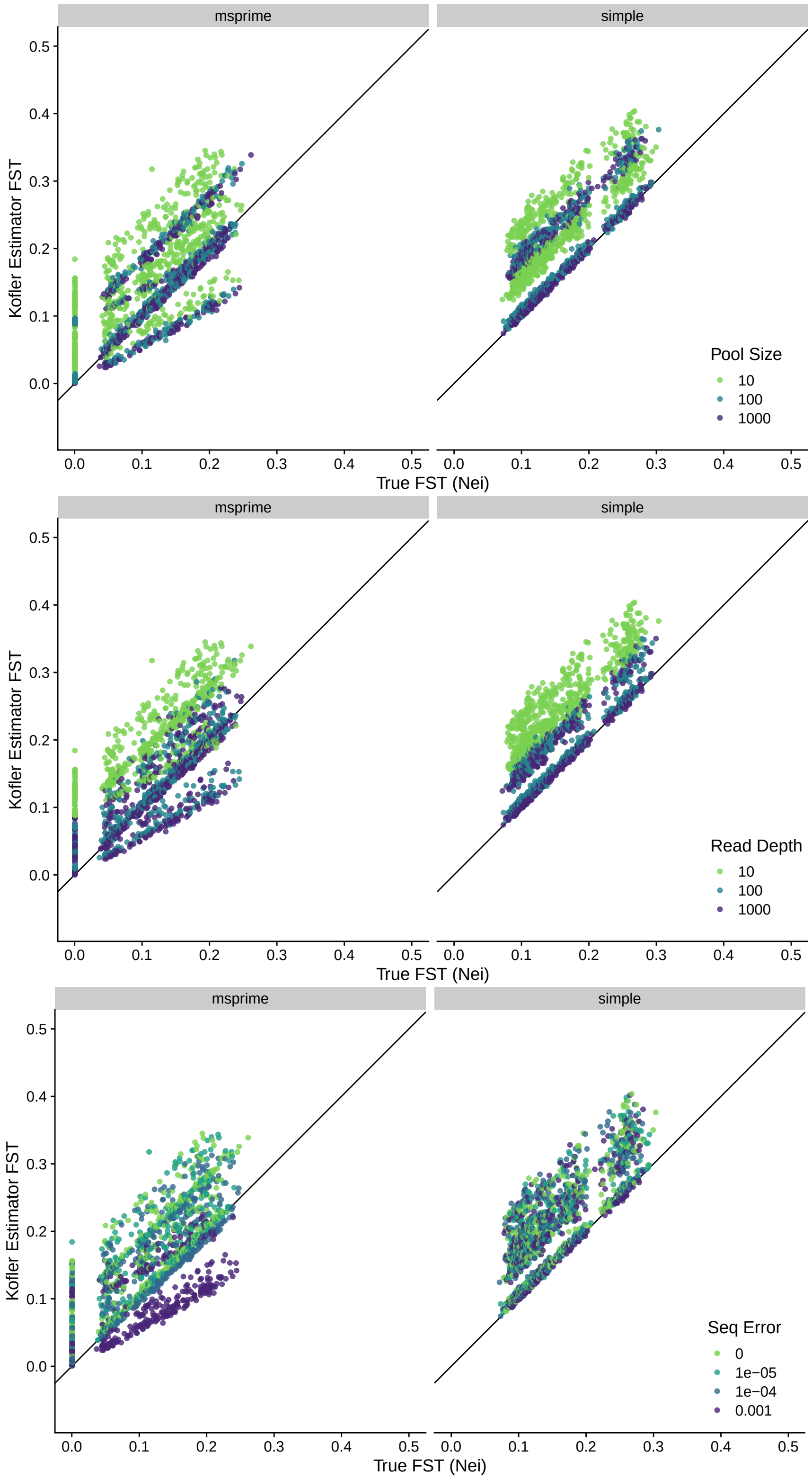

Figure 3: Kofler Estimator for FST vs true FST (Nei).

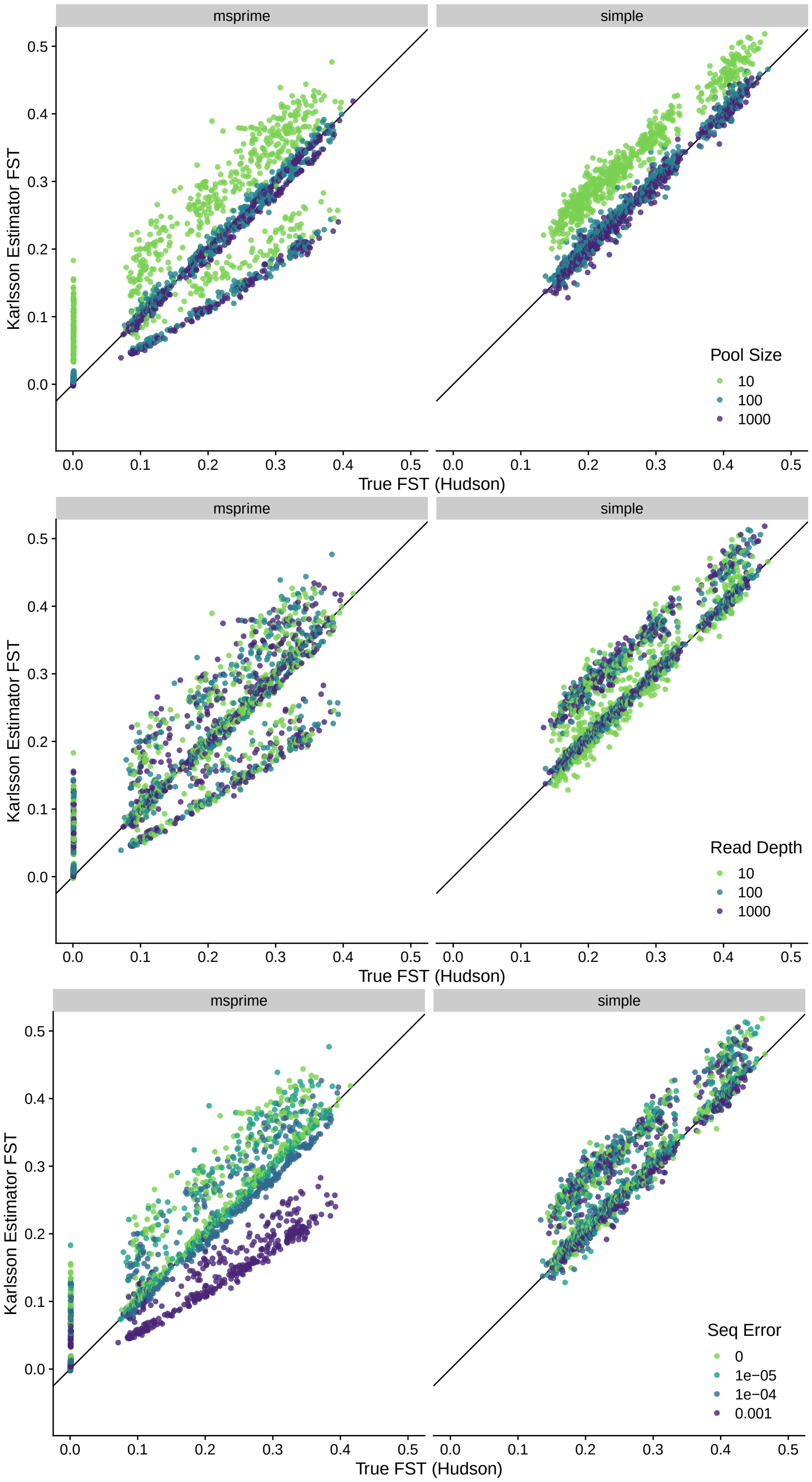

Figure 4: Karlsson Estimator for FST vs true FST (Hudson).

Evaluation and Results

We show the results of the simulations in Figures 1-4. Each Figure shows the comparison of the estimated values versus the true values, for `msprime` on the left, and for the "simple" simulation on the right. Each plot is repeated three times, colorized by Pool Size, Read Depth, and Sequencing Error, respectively, in order to show the effects of each of these parameters on the estimation.

We find that the estimators we derived in Section 5.2 are approximately unbiased across all regimes except for the `msprime` simulations with the highest sequencing error rate, see Figure 1 and Figure 2. In particular, it can be observed that for larger pool sizes, the variance of the estimators reduces, as expected, as well as, to some degree, for higher read depths.

In contrast, in Figure 3 we find that the Kofler estimator of $F_{\text{ST}}^{\text{Nei}}$ is highly biased in a way that depends on pool size and average read depth. This makes sense because as discussed above, the Kofler estimate of π_{between} includes a Bessel correction when it should not. That Bessel correction contains terms that depend on both the pool size and the the read depth, explaining the pool size and sample size-dependent bias.

Similarly, in Figure 4 we find that the Karlsson estimator of $F_{\text{ST}}^{\text{Hudson}}$ is highly biased for small pool sizes, but otherwise performs well (again except for the case of high sequencing error). Again, this makes sense as the Karlsson estimator implicitly assumes an infinite pool size, and so will be biased for small pool sizes.

For the `msprime` simulations with the highest sequencing error rate, we see that all methods severely underestimate F_{ST}. It makes sense that the methods should be biased that way, as none of the estimators account for sequencing error. In the `msprime` simulations, most sites are not segregating in the population. Sites that are not segregating do not contribute to the true value of F_{ST} — they act as a 0 added to each of the π's. Calling the rate of sequencing error ϵ, all of the above estimators add something $\approx \epsilon$ over the number of sites to each of the $\widehat{\pi}$'s in expectation. As a result, for $\widehat{F_{\text{ST}}^{\text{Nei}}} := 1 - \widehat{\pi_{\text{within}}}/\widehat{\pi_{\text{total}}}$, for example, we add something on the order of ϵ to our estimates of $\widehat{\pi}_{\text{within}}$ and $\widehat{\pi}_{\text{total}}$. This inflates $\widehat{\pi_{\text{within}}}/\widehat{\pi_{\text{total}}}$ causing us to underestimate $F_{\text{ST}}^{\text{Nei}}$.

A similar phenomenon occurs for $\widehat{F_{\text{ST}}^{\text{Hudson}}}$, as well as the other estimators. To see that invariant sites add $\approx \epsilon$ over the total number of sites to any of the $\widehat{\pi}$'s, consider an asymptotic regime where both the read depth and the pool size are large. In this regime, if there were no sequencer error, the empirical frequency of each allele across reads is a very accurate estimate of the the true population allele frequency. In particular, for invariant sites, all of the reads will show the "0" allele across both populations. If we add sequencing error, then approximately ϵ proportion of the reads will show the "1" allele in each population. Our estimators as introduced above ignore sequencing error, and so in this case, it would look like both populations have the "1" allele at frequency ϵ. Naively plugging these frequencies into π_{within}, π_{between}, or π_{total} we obtain $\epsilon(1-\epsilon) \approx \epsilon$. In practice, at invariant sites, the different estimators would include various Bessel corrections, but the intuition holds, and these sites contribute approximately ϵ to the overall estimates of the different π's. As a result, if ϵ is comparable to or larger than any of the π's, then any of the estimators will be significantly biased. Indeed, in the `msprime` simulations, we would expect π_{within} to be approximately θ, which is 0.001, matching the highest sequencing error simulations, and explaining the apparent bias. In contrast, in the "simple" simulations, π_{within} is approximately 0.03 which is an order of magnitude larger than the highest sequencing error simulated and hence the bias is negligible.

5.6 Unbiased estimates of heterozygosity in the presence of sequencing error

In the following, we present derivations of the above estimators of F_{ST} that also correct for sequencing errors. This is currently not implemented in our software, and in no other software that we are aware of. Our rationale is that many applications are based on short-read Illumina sequencing, which has an error of 1/1000. We however present it here for completeness, in the hope that this is useful for others, especially if other types of next generation sequencing with higher error rates are used.

Overview

We will derive estimators for π_{within}, π_{between}, and π_{total} in the presence of sequencing error. In the simulations above, and by a hand-wavy theoretical argument, we saw that if sequencing error is on the order of π_{within}, π_{between}, or π_{total}, then our estimates of F_{ST} can be severely biased. We consider two cases: one where we have access to read-level estimates of sequencing error

(e.g., phred scores), and one where we just have access to an estimate of the average sequencing error rate. Since the second is a special case of the first (i.e., we can just assume all reads have the same sequencing error), it will follow straightforwardly but will simplify some equations.

One interesting and important observation to note here is that when we did not account for sequencing error, our estimates of π_{within}, π_{between}, and π_{total} were all zero for monomorphic (non-segregating) sites. This allowed us to sum the estimates of π_{within}, π_{between}, and π_{total} across only segregating sites and then divide by the total number of sites (both segregating and non-segregating). We will see that once we account for sequencing error, monomorphic sites actually contribute a small amount to π_{within}, π_{between}, and π_{total}, as there will now be an *additive* bias correction in addition to a multiplicative one. As such, when computing π_{within}, π_{between}, or π_{total} in a window, we will need to sum across segregating sites and then add a term that is the number of non-segregating sites times the estimate of π_{within}, π_{between}, or π_{total} for a non-segregating sites, and then finally divide that whole expression by the total number of sites.

We will start with our general model, derive our estimators from there, and then show that they are unbiased.

Model

We will follow the notation from above where applicable, but briefly reintroduce some terms for ease of reading. For generality, we will assume that there are K possible alleles; this will allow us to switch between biallelic model and models on $\{A, C, G, T\}$. We let f_τ be the frequency of allele τ *in the population*. Then we let N_τ be the (unobserved) number of individuals in the pool of n individuals with allele τ. Finally, let there be c reads at a site, and let R_j be the allele of the individual sequenced on the jth read, and let $\hat{R}_j$ be the actual output of the sequencer for the jth read. That is, if $R_j = \tau$, then the jth read comes from an individual that has the τ allele, but then once we sequence that fragment, there is a small chance that an error is introduced, so it is very likely that $\hat{R}_j$ — the allele output by the sequencer — is τ if R_j is τ, but there is some chance that the sequencer reads a different allele and then $\hat{R}_j = \tau' \neq \tau$. In the most general case, we will assume that $\hat{R}_j$ matches R_j with probability $1 - \epsilon_j$ and randomly chooses an allele other than R_j with probability ϵ_j and chooses among the options uniformly at random. For example, if we are looking at $\{A, C, G, T\}$ and the sequencing error rate of read j is ϵ_j, then if $R_j = A$, we have that $\hat{R}_j = A$ with probability $1 - \epsilon_j$ and $\hat{R}_j = C$ with probability $\epsilon_j/3$, and $\hat{R}_j = G$ with probability $\epsilon_j/3$, and $\hat{R}_j = T$ with probability $\epsilon_j/3$.

Throughout we will use the following results for $j \neq j'$:

$$\mathbb{E}\left[\mathbb{I}\left\{\hat{R}_j = \tau\right\}\right] = \frac{\epsilon_j}{K-1} + \left(1 - \frac{K}{K-1}\epsilon_j\right) f_\tau \tag{55}$$

$$\begin{aligned}\mathbb{E}\left[\mathbb{I}\left\{\hat{R}_j = \tau\right\}\mathbb{I}\left\{\hat{R}_{j'} = \tau\right\}\right] = &\frac{\epsilon_j \epsilon_{j'}}{(K-1)^2} \\ &+ \frac{1}{n}\left((1-\epsilon_j)(1-\epsilon_{j'}) + \frac{n-1}{K-1}(\epsilon_j + \epsilon_{j'} - 2\epsilon_j\epsilon_{j'}) + \frac{1-2n}{(K-1)^2}\epsilon_j\epsilon_{j'}\right) f_\tau \\ &+ \frac{n-1}{n}\left((1-\epsilon_j)(1-\epsilon_{j'}) - \frac{1}{K-1}(\epsilon_j + \epsilon_{j'} - 2\epsilon_j\epsilon_{j'}) + \frac{1}{(K-1)^2}\epsilon_j\epsilon_{j'})\right) f_\tau^2\end{aligned} \tag{56}$$

Before going any further, these look a bit complex, so let's do a quick sanity check. If $\epsilon_j = 0$, then we get

$$\mathbb{E}_{\epsilon=0}\left[\mathbb{I}\left\{\hat{R}_j = \tau\right\}\right] = f_\tau$$

$$\mathbb{E}_{\epsilon=0}\left[\mathbb{I}\left\{\hat{R}_j = \tau\right\}\mathbb{I}\left\{\hat{R}_{j'} = \tau\right\}\right] = \frac{1}{n}f_\tau + \frac{n-1}{n}f_\tau^2,$$

which it turns out matches what we would get from looking at reads in the model with no error in the equations as described in the sections above. Above, we considered the empirical frequency $\widehat{f_\tau} := \frac{1}{c}\sum_{j=1}^{c}\mathbb{I}\left\{\hat{R}_j = \tau\right\}$, and saw that if there is no sequencing error $\mathbb{E}\left[\widehat{f_\tau}(1-\widehat{f_\tau})\right] = \frac{n-1}{n}\frac{c-1}{c}f_\tau(1-f_\tau)$. We leave it as a straightforward exercise to see that the above (when all ϵ_j are zero) implies the same expectation for $\widehat{f_\tau}(1-\widehat{f_\tau})$.

To see equation (55), note that by the tower property (twice),

$$
\begin{aligned}
\mathbb{E}\left[\mathbb{I}\left\{\hat{R}_j = \tau\right\}\right] &= \mathbb{E}\mathbb{E}\left[\mathbb{I}\left\{\hat{R}_j = \tau\right\}|R_j\right] \\
&= \mathbb{E}\left[(1-\epsilon_j)\mathbb{I}\left\{R_j = \tau\right\}\right] + \mathbb{E}\left[\frac{\epsilon_j}{K-1}\mathbb{I}\left\{R_j \neq \tau\right\}\right] \\
&= (1-\epsilon_j)\mathbb{E}\mathbb{E}\left[\mathbb{I}\left\{R_j = \tau\right\}|N_\tau\right] + \frac{\epsilon_j}{K-1}\mathbb{E}\mathbb{E}\left[\mathbb{I}\left\{R_j \neq \tau\right\}|N_\tau\right] \\
&= (1-\epsilon_j)\mathbb{E}\left[\frac{N_\tau}{n}\right] + \frac{\epsilon_j}{K-1}\mathbb{E}\left[\frac{n-N_\tau}{n}\right] \\
&= (1-\epsilon_j)f_\tau + \frac{\epsilon_j}{K-1}(1-f_\tau) \\
&= \frac{\epsilon_j}{K-1} + \left(1 - \frac{K}{K-1}\epsilon_j\right) f_\tau
\end{aligned}
$$

Equation (56) follows the same reasoning but with substantially more algebra, so we omit it here for simplicity.

One last useful fact is that if we sum equations (55) and (56) over τ, we obtain something slightly nicer, since $\sum_\tau f_\tau = 1$. With this summation and some slight algebraic manipulation we see:

$$
\sum_\tau \mathbb{E}\left[\mathbb{I}\left\{\hat{R}_j = \tau\right\}\right] = 1 \tag{57}
$$

$$
\begin{aligned}
\sum_\tau \mathbb{E}\left[\mathbb{I}\left\{\hat{R}_j = \tau\right\}\mathbb{I}\left\{\hat{R}_{j'} = \tau\right\}\right] = \frac{1}{n}\left((1-\epsilon_j)(1-\epsilon_{j'}) + \frac{n-1}{K-1}(\epsilon_j + \epsilon_{j'} - 2\epsilon_j\epsilon_{j'}) + \frac{(K-2)n+1}{(K-1)^2}\epsilon_j\epsilon_{j'}\right) \\
+ \frac{n-1}{n}\left((1-\epsilon_j)(1-\epsilon_{j'}) - \frac{1}{K-1}(\epsilon_j + \epsilon_{j'} - 2\epsilon_j\epsilon_{j'}) + \frac{1}{(K-1)^2}\epsilon_j\epsilon_{j'}\right) \\
\times \sum_\tau f_\tau^2
\end{aligned} \tag{58}
$$

With these preliminaries out of the way, we can get to our estimators.

Unbiased estimator for π_{within}

Recall that π_{within} is defined as $\frac{1}{2}\left(\pi_{(1)} + \pi_{(2)}\right)$, where $\pi_{(p)}$ is the heterozygosity in population p. Since this is linear, we just need an unbiased estimator for the heterozygosity within one population, and then we can average these estimators across the populations to obtain an estimator of π_{within}.

Our estimator is a bit unwieldy, so first we define a number of additional variables to clean up the notation. Note that all of these only depend upon observable quantities (i.e., the sample size, read depth, and error rates) so they can be readily computed from

the observed data.

$$
\begin{aligned}
\overline{\epsilon} &:= \frac{1}{c}\sum_{j=1}^{c}\epsilon_j \\
\overline{\epsilon^2} &:= \frac{1}{c}\sum_{j=1}^{c}\epsilon_j^2 \\
\epsilon^* &:= (\overline{\epsilon})^2 - \frac{\overline{\epsilon^2}}{c} \\
w_1 &:= \frac{1}{c} + \frac{1}{n}\left(\frac{c-1}{c} + 2\left(\frac{c-1}{c}\right)\left(\frac{n-K}{K-1}\right)\overline{\epsilon} + \left(1 - 2\left(\frac{n-1}{K-1}\right) + \frac{(K-2)n+1}{(K-1)^2}\right)\epsilon^*\right) \\
w_2 &:= \frac{n-1}{n}\left(\frac{c-1}{c} - 2\left(\frac{c-1}{c}\right)\left(\frac{K}{K-1}\right)\overline{\epsilon} + \left(1 + \frac{2}{K-1} + \frac{1}{(K-1)^2}\right)\epsilon^*\right)
\end{aligned}
$$

We are now ready to define our unbiased estimator of π within a given population. Let $\hat{f}_\tau = \sum_{j=1}^{c} \mathbb{I}\left\{\hat{R}_j = \tau\right\}$ be the empirical frequency of allele τ in the observed reads.

$$
\widehat{\pi} := \frac{w_1 + w_2 - \sum_\tau \hat{f}_\tau^2}{w_2}.
$$

It is a bit of work to show, but it turns out that $\mathbb{E}[\widehat{\pi}] = \pi$. Hence, this estimator is an unbiased estimator for the heterozygosity within a population. The overall proof is to show that

$$
\sum_\tau \mathbb{E}\left[\hat{f}_\tau^2\right] = w_1 + w_2 \sum_\tau f_\tau^2, \tag{59}
$$

which we will show below, but for now we assume this being true. Then, we can use this formula to see

$$
\mathbb{E}\left[\widehat{\pi}\right] = \frac{w_1 + w_2 - \sum_\tau \mathbb{E}\left[\hat{f}_\tau^2\right]}{w_2} = 1 - \sum_\tau f_\tau^2 =: \pi
$$

as required.

Now to see equation (59), we write

$$
\begin{aligned}
\sum_\tau \mathbb{E}\left[\hat{f}_\tau^2\right] &= \frac{1}{c^2}\sum_{j=1}^{c}\sum_{j'=1}^{c}\sum_\tau \mathbb{E}\left[\mathbb{I}\left\{\hat{R}_j = \tau\right\}\mathbb{I}\left\{\hat{R}_{j'} = \tau\right\}\right] \\
&= \frac{1}{c^2}\left\{\left(\sum_{j=1}^{c}\mathbb{E}\left[\mathbb{I}\left\{\hat{R}_j = \tau\right\}\right]\right) + \left(\sum_{j\neq j'}\mathbb{E}\left[\mathbb{I}\left\{\hat{R}_j = \tau\right\}\mathbb{I}\left\{\hat{R}_{j'} = \tau\right\}\right]\right)\right\} \\
&= w_1 + w_2\sum_\tau f_\tau^2
\end{aligned}
$$

where the final line follows from using Equations (57) and (58) and then doing quite a bit of algebra.

Unbiased estimator for π_{between}

Fortunately, the estimator for π_{between} is much simpler. From equation (55), we see

$$\mathbb{E}\left[\hat{f}_\tau\right] = \frac{1}{c}\sum_{j=1}^{c}\mathbb{E}\left[\mathbb{I}\left\{\hat{R}_j = \tau\right\}\right]$$

$$= \frac{1}{c}\sum_{j=1}^{c}\frac{\epsilon_j}{K-1} + \left(1 - \frac{K}{K-1}\epsilon_j\right) f_\tau$$

$$= \frac{\overline{\epsilon}}{K-1} + \left(1 - \frac{K}{K-1}\overline{\epsilon}\right) f_\tau$$

Rearranging, this immediately implies that

$$\frac{\hat{f}_\tau - \frac{\overline{\epsilon}}{K-1}}{1 - \frac{K}{K-1}\overline{\epsilon}}$$

is unbiased for f_τ. So far we have suppressed the dependence of all of these things on the population (i.e., we've just been looking at a single sample), but for π_{between} we must consider two samples. We will use a (p) superscript to denote the population. Our estimator for π_{between} is then

$$\widehat{\pi}_{\text{between}} := 1 - \sum_\tau \left(\frac{\hat{f}_\tau^{(1)} - \frac{\overline{\epsilon}^{(1)}}{K-1}}{1 - \frac{K}{K-1}\overline{\epsilon}^{(1)}}\right)\left(\frac{\hat{f}_\tau^{(2)} - \frac{\overline{\epsilon}^{(2)}}{K-1}}{1 - \frac{K}{K-1}\overline{\epsilon}^{(2)}}\right)$$

That this is unbiased follows immediately from the unbiasedness of our estimators of f_τ within each population and the independence of the two samples.

Unbiased estimator for π_{total}

Recall that $\pi_{\text{total}} = \frac{1}{2}\left(\pi_{\text{within}} + \pi_{\text{between}}\right)$ so with our unbiased estimators of π_{within} and π_{between}, we immediately by the linearity of expectation obtain that:

$$\widehat{\pi}_{\text{total}} := \frac{1}{2}\left(\widehat{\pi}_{\text{within}} + \widehat{\pi}_{\text{between}}\right)$$

is unbiased for π_{total}.

Constant error rates

If we assume that all reads have the same error rates, say ϵ, then $\overline{\epsilon} = \epsilon$, $\overline{\epsilon^2} = \epsilon^2$, and $\epsilon^* = \frac{c-1}{c}\epsilon^2$. Otherwise, the equations remain unchanged.

6 PoPoolation Equations Document

The PoPoolations equation document also presents some simplifications and related equations that to the best of our knowledge are not implemented in their software. We hence do not go through them in detail here, but still want to mention them, in case they might be useful for others.

- They present simplified versions of θ_π, θ_w, and Tajima's D, which assume that allele frequency distribution in the reads is about the same as in the real population, and hence arrives at a simpler computation at the cost of some error. These are also useful for individual sequencing.
- As mentioned above in Section 4.1, the document presents an approach to computing Tajima's D based on its variance, and extends this to windows, but (to the best of our knowledge) does not implement this, and instead implement their approach based on Achaz (2008) [1].

- They present an approach for computing F_{ST} for J pool-sequenced populations (instead of just two as presented above), extend this approach to large regions as well as single SNPs, and introduce weights that take the number of sequenced individuals in each population into account. More work is needed to compare this approach to their implementation and to our estimators.

These alternative approaches however need further assessment and comparison to the other approaches presented here.

7 References

[1] Achaz, G. (2008). Testing for neutrality in samples with sequencing errors. *Genetics*, **179**(3), 1409–1424.

[2] Baumdicker, F. *et al.* (2022). Efficient ancestry and mutation simulation with msprime 1.0. *Genetics*, **220**(3), iyab229.

[3] Bhatia, G. *et al.* (2013). Estimating and interpreting FST: The impact of rare variants. *Genome Research*, **23**(9), 1514–1521.

[4] Bickel, P. J. and Doksum, K. A. (1977). *Mathematical Statistics: Basic Ideas and Selected Topics*. Holden-Day Series in Probability and Statistics. Prentice Hall.

[5] Cockerham, C. C. (1969). Variance of Gene Frequencies. *Evolution*, **23**(1), 72.

[6] Ferretti, L. *et al.* (2013). Population genomics from pool sequencing. *Molecular Ecology*, **22**(22), 5561–5576.

[7] Gautier, M. *et al.* (2022). f-statistics estimation and admixture graph construction with Pool-Seq or allele count data using the R package poolfstat. *Mol. Ecol. Resour.*, **22**(4), 1394–1416.

[8] Hahn, M. W. (2018). *Molecular Population Genetics*. Sinauer Associates, Oxford University Press.

[9] Hartl, D. L. and Clark, A. G. (2007). *Principles of Population Genetics*. Sinauer.

[10] Hivert, V. *et al.* (2018). Measuring Genetic Differentiation from Pool-seq Data. *Genetics*, **210**(1), 315–330.

[11] Hudson, R. R. *et al.* (1992). Estimation of levels of gene flow from DNA sequence data. *Genetics*, **132**(2), 583–589.

[12] Karlsson, E. K. *et al.* (2007). Efficient mapping of mendelian traits in dogs through genome-wide association. *Nature Genetics*, **39**(11), 1321–1328.

[13] Kelleher, J. *et al.* (2016). Efficient coalescent simulation and genealogical analysis for large sample sizes. *PLoS computational biology*, **12**(5), e1004842.

[14] Kofler, R. *et al.* (2011a). PoPoolation: A Toolbox for Population Genetic Analysis of Next Generation Sequencing Data from Pooled Individuals. *PLoS ONE*, **6**(1), e15925.

[15] Kofler, R. *et al.* (2011b). PoPoolation2: identifying differentiation between populations using sequencing of pooled DNA samples (Pool-Seq). *Bioinformatics*, **27**(24), 3435–3436.

[16] Nei, M. (1973). Analysis of Gene Diversity in Subdivided Populations. *Proceedings of the National Academy of Sciences*, **70**(12), 3321–3323.

[17] Weir, B. S. and Hill, W. G. (2002). Estimating F-Statistics. *Annual Review of Genetics*, **36**, 721–750.
