# Supplementary material for: grenedalf: population genetic statistics for the next generation of pool sequencing
Source: Bioinformatics. 2024 Aug 26;40(8):btae508. doi: 10.1093/bioinformatics/btae508 (PMC11357794; doi:10.1093/bioinformatics/btae508)
Supplement: btae508_Supplementary_Data [file btae508_supplementary_data.zip › Software Comparison. Benchmarking the computational requirements of grenedalf versus existing tools.pdf]

Supplementary Text: Software Comparison

grenedalf: population genetic statistics for the next generation of pool sequencing

Lucas Czech[a,b,*], Jeffrey P. Spence[c], and Moisés Expósito-Alonso[a,d,e,f,g*]

[a]Department of Plant Biology, Carnegie Institution for Science, Stanford, USA.
[b]Section for GeoGenetics, Globe Institute, University of Copenhagen, Denmark.
[c]Department of Genetics, Stanford University, Stanford, USA.
[d]Department of Biology, Stanford University, Stanford, USA.
[e]Department of Global Ecology, Carnegie Institution for Science, Stanford, USA.
[f]Department of Integrative Biology, University of California Berkeley, Berkeley, USA.
[g]Howard Hughes Medical Institute, University of California Berkeley, Berkeley, USA.

[*]To whom correspondence should be addressed:
lczech@carnegiescience.edu, and moisesexpositoalonso@gmail.com

In this supplement, we compare GRENEDALF to existing software for computing population genetics statistics for Pool-seq data, and elaborate on some of the computational improvements that GRENEDALF offers. We evaluate the runtime and memory requirements for some representative and frequently performed tasks by comparing GRENEDALF to libraries and tools that have similar scope and functionality. Note that GRENEDALF internally uses our software library GENESIS [1], meaning that the benchmarks shown here are applicable to both.

Contents

1 Existing Tools 2

2 File Formats 3

3 Benchmarks and Comparisons 4

4 References 25

1 Existing Tools

We compare GRENEDALF to the following existing tools:

- POPOOLATION [2] computes the diversity statistics θ_π, Watterson's θ, and Tajima's D, is written in Perl, and takes `pileup` files as input. Input is traversed in windows of a given interval size, and is streamed, keeping the memory usage low.

- POPOOLATION2 [3] computes population differentiation with F_{ST}, is also written in Perl, and takes their ad-hoc `sync` file as input. It also works in interval windows and streams through the data. Note that despite the naming of the tool implying it to be an update to POPOOLATION, it is in fact a distinct stand-alone tool.

- POOLFSTAT [4, 5] computes F_{ST} (as well as some other f- and D-statistics, which we have not yet implemented in GRENEDALF, and hence ignore for the time being), and is written in C++ and R. Its input format is also `sync`. Due to using R datatypes, it reads all input into memory at once. It computes F_{ST} in windows between pairs of samples, and also has a function to compute F_{ST} between multiple samples at once, but only for the whole genome.

- NPSTAT [6] computes several diversity metrics, and is written in C. It takes `mpileup` as input, and computes statistics in windows of a fixed number of bases; it can however only compute data from one chromosome at a time.

- SAMTOOLS [7] is a toolkit for working with `sam`/`bam` and `(m)pileup` files. It does not compute any population genetic statics that we are interested in here, but we use it here as a baseline for benchmarking tasks such as file conversions.

In GRENEDALF, we implemented all the file formats that the above tools offer, as well as the considerably more complex `sam` (and its gzipped form `sam.gz`), `bam`, and `cram` files, which are what typical read mapping tools produce, and for which we internally use HTSLIB [8]. In many cases, this hence eliminates the need for *a priori* file conversions. Furthermore, we offer to read from `vcf` files [9] by utilizing their `AD` format field (allelic depth, which is relevant when genotypes are heterozygous, polyploid, or multiple genotypes are present in a sample such as in in Pool-seq). Lastly, we can read from a variety of allele frequency table formats, similar to what is produced by HAF-PIPE [10, 11] or other allele frequency estimators. To the best of our knowledge however, these file formats are not standardized for Pool-Seq data, and not implemented in any other tool; we hence do not include benchmarks based on these formats in our comparison to other tools here.

Remark on terminology: In order to avoid confusion, we have decided to use the term "read depth" throughout to mean the number of reads that are present at a particular position in the genome. This is opposed to the ambiguous term "coverage", which is instead used by PoPoolation and npstat. However, this might lead to confusion about whether this is meant to indicate "coverage depth" (that is, "read depth") or "coverage breadth". Ideally, we would even want to distinguish the average depth (e.g., 30X "coverage depth"), from the specific "read depth" at a particular locus. As we only have the latter use case here, we have hence decided to simply use "read depth" throughout.

2 File Formats

We here give a brief introduction of the file formats that GRENEDALF can read (at the time of writing this document), for the reader's convenience.

- `sam`/`bam`/`cram` [7]: Widely-used standard for storing and representing sequence alignment data, i. e., reads aligned to a reference genome, with `sam` being the text-based format and `bam` being its binary equivalent, and `cram` being a further compressed variant. Data is stored per-read, meaning that for the computations that we do here, we first need to construct an internal per-position representation based on reads overlapping each position.

- `(m)pileup` [7]: Representation of the bases at each genomic position, where each line "piles up" the bases found across all reads that span that position. A multi-pileup (indicated by the `m` in the name) contains multiple samples, piled up on the same line in the file. This format hence already contains the data in the orientation that we need it here, but comes at the expense of extra disk space and time needed for the conversion from `sam`/`bam`. Thus, it usually does not make sense to convert to pileup when working with GRENEDALF.

- `sync` [3]: A simple tab-delimited text format, where each line represents a specific genomic position and includes allele counts for multiple populations, for each of the four bases. As it only contains simple counts, it is very efficient to parse, but unfortunately (in its original form) does not support further data annotation, or even sample names. Our implementation in GRENEDALF supports some (semi-established) *ad-hoc* extensions such as sample names and masked positions, which help with bookkeeping during analyses.

- `vcf` [9]: Widely used file format that represents genetic variants per position, such as single nucleotide polymorphisms (SNPs) and insertions/deletions (indels), including their alleles, quality scores, and additional annotations. In pool sequencing, the AD (Allelic Depth) field can be utilized to represent the read depth or allele counts for each allele in a pool of sequenced individuals. We however do not recommend using `vcf` data coming from variant callers (e. g., GATK) directly applied to pool sequencing data, as this likely introduces bias in frequencies [12].

- Frequency tables: This is not a specific format, but simply any generic table format with positions along the genome in rows, and one or more columns for samples, containing allele frequencies or counts for the given position. Such tables are for example produced by HAF-PIPE [10, 11], and we can read any such format, as long as it contains a header line in a format that we can parse.

When using GRENEDALF to run some simple analyses, we recommend directly running on `bam` files that come out of the alignment step, such as when using GRENEPIPE [13]. This is the easiest way, which avoids file conversions. However, when using GRENEDALF for some more involved analyses, where files have to be read multiple times, we instead recommend converting the `bam` files to `sync` files first. They are substantially faster to process, hence leading to overall speed improvements.

3 Benchmarks and Comparisons

We benchmarked the runtime and memory requirements of GRENEDALF in comparison to existing tools for several typical tasks, as well evaluated the resulting data in terms of consistency and compatibility with other tools. The results are shown in the figures below.

Unless noted otherwise, the tests were run on a laptop computer with an 8-core AMD Ryzen 7 Pro processor (with hyper-threading, for a total of 16 hardware threads), and 32 GB of main memory, running Ubuntu 20.04.5 LTS. We note that GENESIS and GRENEDALF offer multi-threaded processing. They can, for example, read/parse multiple files simultaneously on distinct compute cores and parallelize computations of statistics. We are not aware that any of the competing libraries and tools evaluated here offer this feature. Hence, for fairness, we here also ran GRENEDALF on a single core only where possible for the tool comparisons. Note though that further speedups compared to the ones shown here can be achieved when leveraging the multi-threading, as shown below as well.

Through the comprehensive tests and figures below, we showcase that GRENEDALF consistently and significantly outperforms *all* other tools, is more memory-efficient (or at least not significantly worse), and never uses a prohibitively large amount of memory even for large dataset sizes. As we emphasized, the command line interface for statistics, file manipulations, and utility routines it is considerably more user-friendly than all other software, with comprehensive options for input file formats, filtering, subsetting, windowing approaches, and more.

In particular, the most salient points of comparison of each of the existing tools are as follows:

- PoPoolation/PoPoolation2: Both are generally slow (see, e. g., Figure 4 and Figure 5), and do not offer a whole-genome mode; they would need to read the whole input into memory (Figure 8). Both tools only work with a single input file format (`pileup`, and `sync`, respectively), both of which need prior conversion and, in case of `pileup`, take up large amounts of disk space.
- Poolfstat: Reads the whole input into memory, which is prohibitive for large datasets (Figure 5). It does not seem to report per-SNP values with their respective positions, making it hard to track where each F_{ST} value comes from, and does seem to be able to compute pairwise F_{ST} in windows along the genome.
- npstat: Also needs the large `pileup` format as input, but only processes the first chromosome of the input (Figure 4), and produces invalid results afterwards. It pre-computes the pool-sequencing correction denominators for *all* read depths up to the given maximum, instead of only computing the values based on what is actually observed in the data. This makes it prohibitively slow when working with high depth data. It however computes several other population genetic statistics as well that we have not yet implemented in GRENEDALF.

Below, we show several benchmarks and case studies, evaluating runtime and memory requirements in detail:

- Figure 1 and Figure 2: Exemplary plots based on computing F_{ST}, showing what the improved speed of GRENEDALF can be used for.
- Figure 3, Figure 4, Figure 5: Benchmarks based on real world data, as a showcase with realistic data and assumptions.
- Figure 6, Figure 7, Figure 8: Benchmarks based on randomly simulated data, as a baseline test with very simple input, showing the minimally expected improvement provided by GRENEDALF.

- Figure 9 and Figure 10: Scaling efficiency of GRENEDALF when using multi-threading.
- Figure 11 and Figure 12: Comparison of the diversity estimators (Theta Pi, Theta Watterson, and Tajima's D) between GRENEDALF and PoPOOLATION, based on real world data.
- Figure 13, Figure 14, Figure 15: Comparison of the diversity estimators (Theta Pi, Theta Watterson, and Tajima's D) between GRENEDALF and NPSTAT, based on real world data.
- Figure 16 and Figure 17: Comparison of the Fst estimators between GRENEDALF and PoPOOLATION2, based on real world data.
- Figure 18 and Figure 19: Evaluation of the biases of different estimators of F_{ST} on real world data, corroborating our findings on simulated data as shown in our assessment of the equations.

The scripts for running the tests and creating the plots shown here are provided at https://github.com/lczech/grenedalf-paper.

Additionally, in order to double-check the correctness of our implementation of the equations, we also re-implemented a minimal version of all estimators in Python, based on our equations document. This independent implementation follows the document closely, and was used for simulated test under different read depth and pool size conditions. We find that results obtained with our implementation in grenedalf match this simple independent implementation, confirming that the equations are implemented as described.

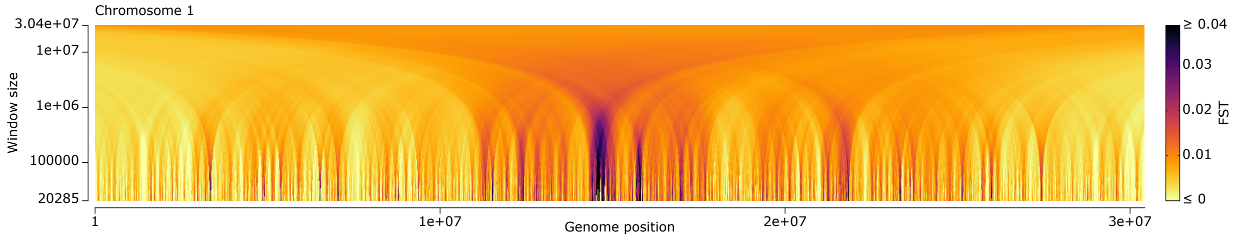

Figure 1: Cathedral plot of F_{ST} between two pool sequencing samples of *A. thaliana*. The faster speed of GRENEDALF allows us to create plots that are computationally more involved. Here, we created what we call a "cathedral plot" of F_{ST} on chromosome 1 between two pool sequencing samples of *A. thaliana*, which reveals the differentiation structure between two populations across different scales, with darker regions having a higher F_{ST} between the two populations.

The x-axis corresponds to chromosome 1 of *A. thaliana*, which is ≈30mio bases long. The y-axis position (rows of pixels) determines the window size to be used for that row. From top to bottom, windows get smaller, with an exponentially decaying size. The top row corresponds to windows of the full length of the chromosome, and towards the bottom, windows get smaller until their size matches the resolution of the image. Here, we plotted the image with a width of 1500 pixels, so that each pixel in the bottom row represents ≈20k positions in the genome. For a given image width, this is the highest resolution that can be displayed; at this point, each window is exactly as wide as the pixel it corresponds to, and windows between pixels in the row are not overlapping with each other any more. Windows are centered around their pixel, meaning that towards the left and right of the plot (and in particular towards the top of the image, with larger window sizes), the actual number of positions within a window is truncated.

In other words, for each pixel, the plot involves accumulating values in a window centered on that pixel, with the width of the window determined by the row in the image. Each pixel hence corresponds to a distinct window width and position, and shows the F_{ST} value in that window. Our implementation computes intermediate per-SNP values once; for F_{ST} for example, we compute π_{within}, π_{between}, and π_{total} for each SNP, as explained in our equations document. Per window, these values are then accumulated and the resulting F_{ST} is computed.

The plot hence "zooms in" from larger to smaller windows, with increasing resolution along the genome towards the bottom. This can help to visualize the scale of a statistic across different window sizes, showing its broad and fine structure, and allows picking appropriate window sizes for further analyses. Note that we chose exponential decay for the window size, as it offers a good balance between revealing both broad structures and fine details. This results in the y-axis being log-scaled. Other functions for the window are however also possible. Of course, similar types of plots can also be useful for other per-window statistics, such as diversity.

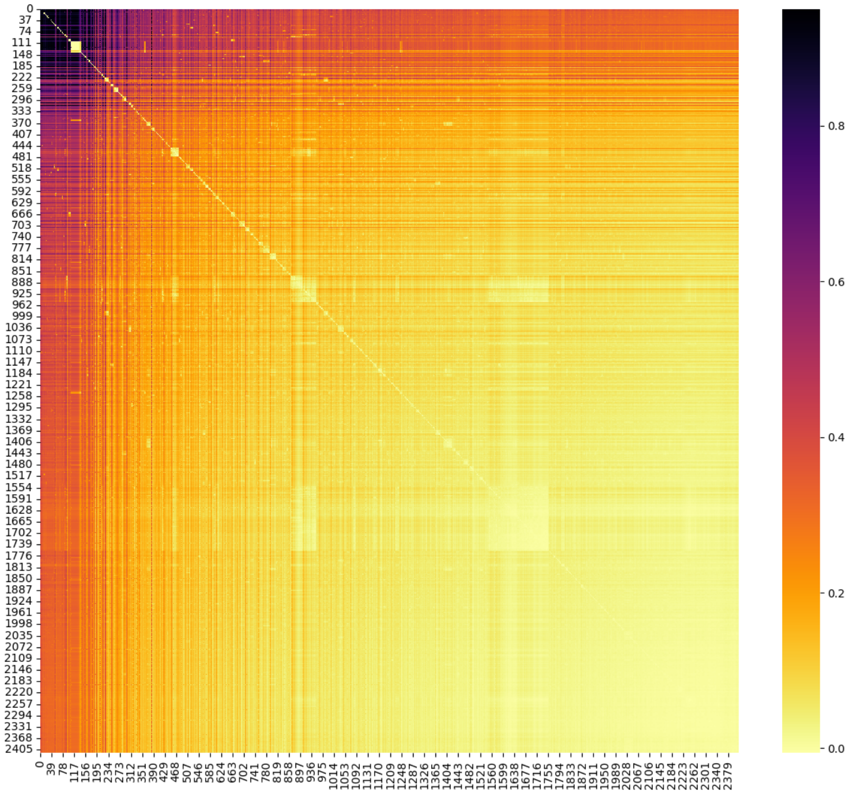

Figure 2: Pairwise whole-genome F_{ST} matrix between 2415 pool sequencing samples. Here, we computed a pairwise F_{ST} matrix between 2415 whole-genome Pool-Sequencing samples of *A. thaliana* from our GrENE-net project, a large-scale Evolve&Resequence project (data not published yet). The input data here consists of 136 GB of frequency tables estimated with HAF-PIPE [10], which for ease of processing were converted into 12 GB of compressed `sync.gz` files. The files cover all five chromosomes of *A. thaliana*, and contain a total of 2,948,475 variant positions.
For all 2,914,905 pairs of samples ($2415 \cdot 2414 \div 2$), we then computed whole-genome F_{ST}, across all variant positions, for a total of ≈8.5 trillion single-SNP F_{ST} computations. We here show the resulting values as a symmetrical heat map; the rows and columns are sorted using average linkage clustering, to make it visually more appealing. We do not further analyse this data here, but simply show it as a use case of the efficiency of GRENEDALF.
In total, the matrix took 16h 20min to compute, and used about 12 GB of memory (mostly due to data buffering to increase efficiency, but this can be reduced if needed). We ran this on a computer cluster, as this is where the data was stored, using 8 threads on the compute node. We further used this test case for measuring thread scaling performance, see Figure 10.
We also tried to get PoPOOLATION2 to run on this dataset (in single SNP mode though, as it would otherwise need a prohibitive amount of memory, due to its caching of all data in a window), but it failed to produce any output even after several hours of waiting. From extrapolation on subsets, we however estimate that it would take about 10 years of computation for it to produce this matrix. If it were to be run with a window size covering the whole genome, in order to obtain a single F_{ST} value per pair of samples as shown here, it would require about 400 TB of memory.

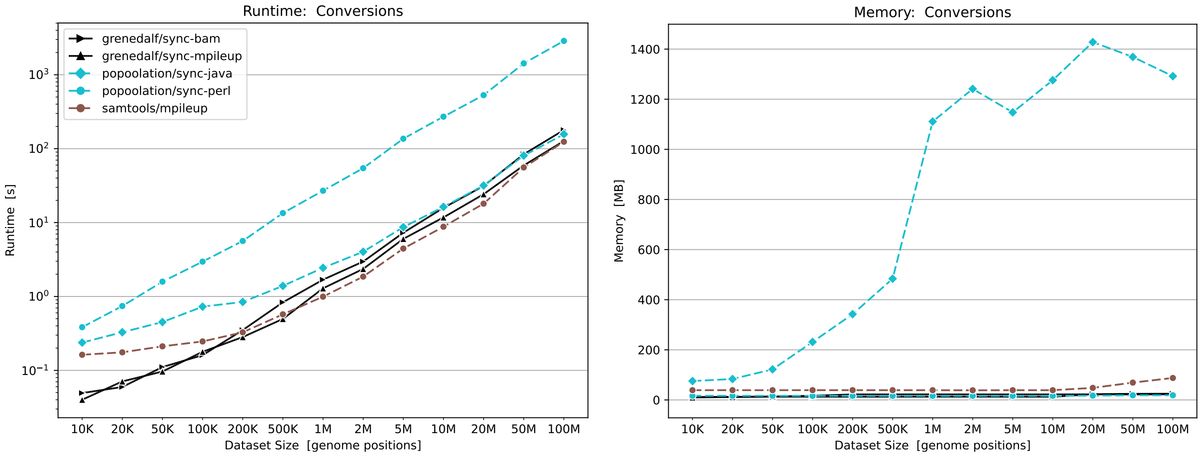

Figure 3: Runtimes (log-log scale) and memory usage (linear–log scale) for converting input `bam` files to the intermediate file formats required by existing tools, for dataset sizes of 10 K to 100 M genomic positions, on real-world data.

Here, we show the computational requirements for the two-step process from `bam` via `(m)pileup` to `sync`, which is typically needed when using PoPoolation2 (for F_{ST}) or poolfstat; PoPoolation (for diversity estimates) only requires the former, i. e., `pileup` files. The processes typically uses samtools for the first, and one of two variants of the PoPoolation sync converter for the second step (written in Java, and Perl, respectively). We also show our implementations in grenedalf, which can produce `sync` files directly from `sam`/`bam` files (without the intermediate `pileup` step), and as well from `(m)pileup` files. In summary, the plot shows all tools that produce a `sync` file from either `bam` or `pileup` input.

The input files consist of subsets of real Pool-Seq samples from our GrENE-net experiment, with the largest `bam` file (containing 100 M genome positions) being about 800 MB in size, its corresponding `pileup` file being 4.0 GB, and the resulting `sync` file being 2.4 GB. Note that file sizes of formats with per-read information such as `sam`/`bam` and `(m)pileup` depend on the number of reads, and are hence determined by genome size and read depth, while accumulated formats such as the `sync` format sum up nucleotide counts per position, meaning that their size only depends on genome size.

These file conversions add overhead to the analysis, in terms of user effort for running the conversions and the associated file management, as well as the additional disk space needed. For example, for a typical Pool-Seq sample of *A. thaliana* of our GrENE-net dataset, an extra ≈8.1 GB are needed for the two files, given an input `bam` file of 1.0 GB. Hence, in grenedalf, we also implemented to read directly from (coordinate-sorted) `sam`/`bam`/`cram` files, which eliminates the need for these file conversions. This comes at the expense of a slight increase in runtime due to the more complex file format (and its dependency on read depth), as can be seen in the subsequent figures, which is however compensated by the generally faster runtime of grenedalf overall, as well as offset by the saving in time needed for the file conversions in the first place. For example, the runtime overhead for converting from `bam` to `pileup` to `sync` with existing tools is already larger than reading the `bam` file in grenedalf directly.

Still, it might be advantageous to use grenedalf to convert from `sam`/`bam` to `sync`, when the file is going to be read many times, or in order to combine multiple samples into one file, and make subsequent reading even faster. Note also that grenedalf can produce and read gzipped files, bringing down the extra disk space needed for a `sync.gz` file of a typical *A. thaliana* Pool-Seq sample to about 430 MB, with almost the same performance as uncompressed `sync` files, due to decompression being executed asynchronously.

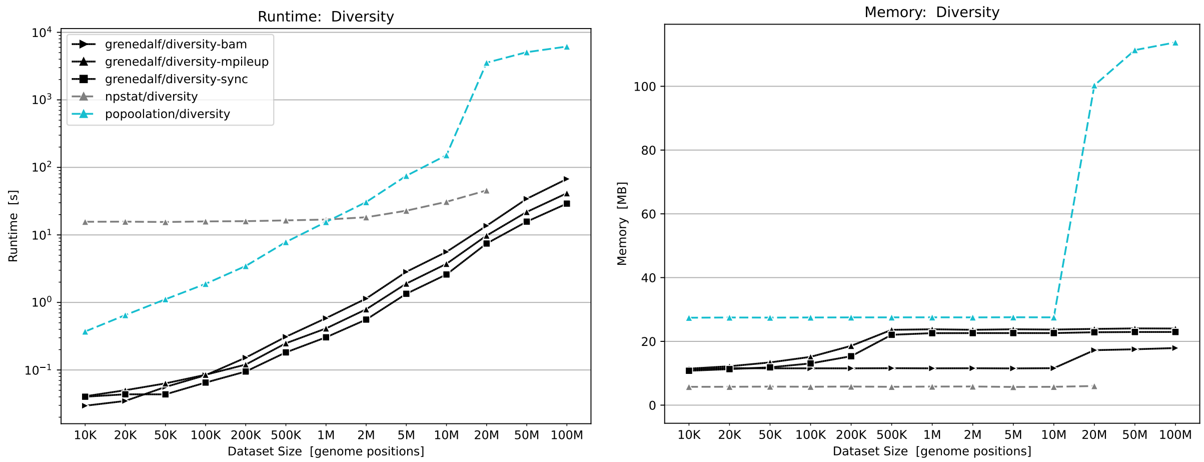

Figure 4: Runtimes (log-log scale) and memory usage (linear–log scale) for computing diversity measures, for dataset sizes of 10 K to 100 M genomic positions, on real-world data. The data are the same as in Figure 3; for GRENEDALF, we also show differences in speed when using different input file formats. PoPoolation and NPSTAT both require `pileup` files for this analysis. Unfortunately, NPSTAT can only process single chromosomes at a time, which here means that we could not run tests beyond 20M base pairs, after which the second chromosome starts in the *A. thaliana* genome.
For all tools, we here computed Tajima's D, in windows of 1000 base pairs. The surge in runtime and memory of PoPoolation when processing 20M or more genome positions is due to characteristics of the dataset: The equations for computing the Pool-Seq bias correction term are dependent on the read depth of the data at a given position, requiring repeated evaluation of a binomial distribution (to be precise: the distribution is evaluated $c \times p$ times, with c the read depth at the position, and p the pool size of the sample). After 20M positions in our exemplary dataset, we first encounter higher read depth positions, hence the surge. Both PoPoolation and GRENEDALF cache the correction terms, to only compute them once per read depth level. In PoPoolation however, an additional intermediate term of the computation is cached, leading to a significant increase in memory consumption. In GRENEDALF, we avoided this extra cache, as it does not increase speed, thus eliminating most of the cache memory requirements. This also explains the flattening of the runtime for PoPoolation, as later on in the data, most read depth values have already been encountered, so that their correction terms are already cached.
Note however that we here limited the maximum read depth to 1000 (see also below), in order to keep runtimes manageable for testing. With higher read depth allowed, PoPoolation would slow down even more, if there are positions in the data with extremely high read depth. On the other hand, the runtime of GRENEDALF does not suffer significantly from this, as our computation of the correction term and the involved binomial distribution is highly optimized (and replaces an expensive summation loop of PoPoolation by a fast closed-form expression, as detailed in our equations document). For instance, we use fast lookup tables of log factorials as well as Stirling's approximation to compute the binomials. Here, that means, on a single core, for the 20M position sample in `pileup` format, GRENEDALF is 380 times as fast as PoPoolation; for the 100M position sample, with the fixed maximum read depth of 1000, it is still more than two orders of magnitude as fast. When reading the faster `sync` format in GRENEDALF instead, the 20M position sample runs 600 times as fast as PoPoolation. This also indicates that the runtime of GRENEDALF is not dominated by the computation of the correction terms.
Lastly, NPSTAT instead pre-computes the Pool-seq correction terms. This is indicated by the near constant runtime for smaller files, as the amount of pre-computation is always the same, and only depends on the specified maximum read depth specified by the user. This is a severe downside of the tool; we had to limit the maximum read depth to 1000 here (for all tools, to keep it comparable), as otherwise NPSTAT would have spend unreasonably long for just the pre-computation. This is not an issue for PoPoolation and GRENEDALF however, which only compute the correction terms as needed in the data.

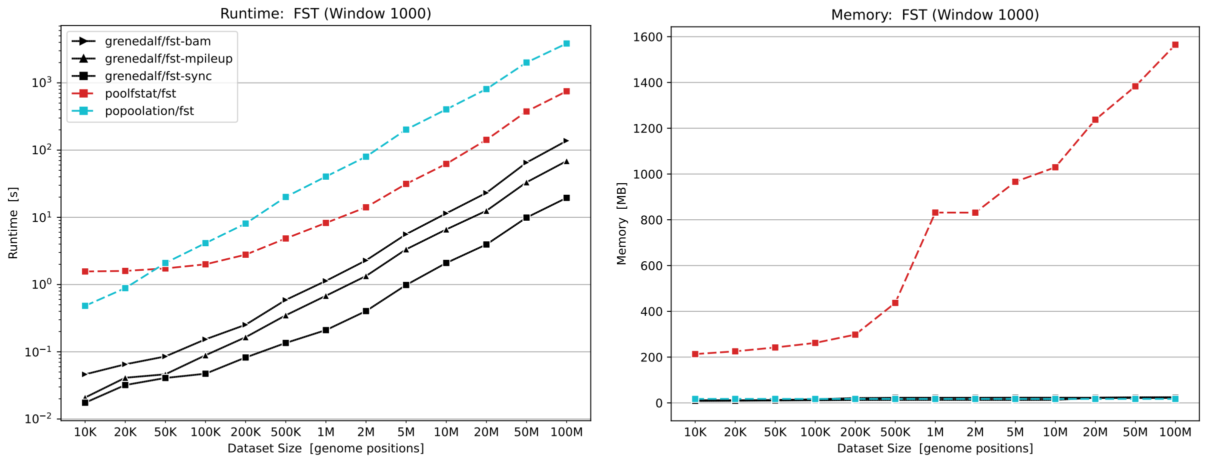

Figure 5: Runtimes (log-log scale) and memory usage (linear–log scale) for computing F_{ST}, for dataset sizes of 10 K to 100 M genomic positions, on real-world data.
We again use data from the same real world experiment as in Figure 3; here however, we use two distinct samples, between which F_{ST} is computed. For GRENEDALF, we again show the benchmark for different input file formats for comparison, with two `bam` files of two samples from GrENE-net, as well as `mpileup` and `sync` files obtained from converting and combining these two `bam` files. The `sync` file is then also used for the two tools we benchmark against here.
The runtime is again best for GRENEDALF using `sync` input files, being about 30 to 40 times faster than POOLFSTAT, and around 200 times faster than PoPOOLATION2. Using more involved input formats which increase in size with read depth, such as `bam` and `mpileup`, slows down GRENEDALF compared to the `sync` format, but it still maintains its advantage over the other two tools.
The way that POOLFSTAT is written in `R`, it requires the input to be fully read into memory before computing F_{ST}. For the largest input file here (with 100 M genomic positions) this equates to ≈1.5 GB memory for the two samples between which we compute F_{ST} here. Note that this requirement linearly scales with the genome size *and* with the number of samples, making POOLFSTAT not feasible for larger datasets with many samples. On the other hand, both PoPOOLATION2 and GRENEDALF stream through the data, so that only data in the order of the window size is needed to be kept in memory; here, with windows of 1000 bps, memory below 25 MB is needed.
This is a particular downside of POOLFSTAT, and prohibits its usage for larger number of samples. For instance (not shown here), we computed pairwise F_{ST} between an increasing number of samples (ranging from 2 to 5 samples) from GrENE-net with POOLFSTAT. Per sample added (each similar in size to the ones used here), the memory requirement of POOLFSTAT increased linearly by about 800 MB. For creating a plot similar to Figure 2, the tool would hence roughly need 2 TB of memory.

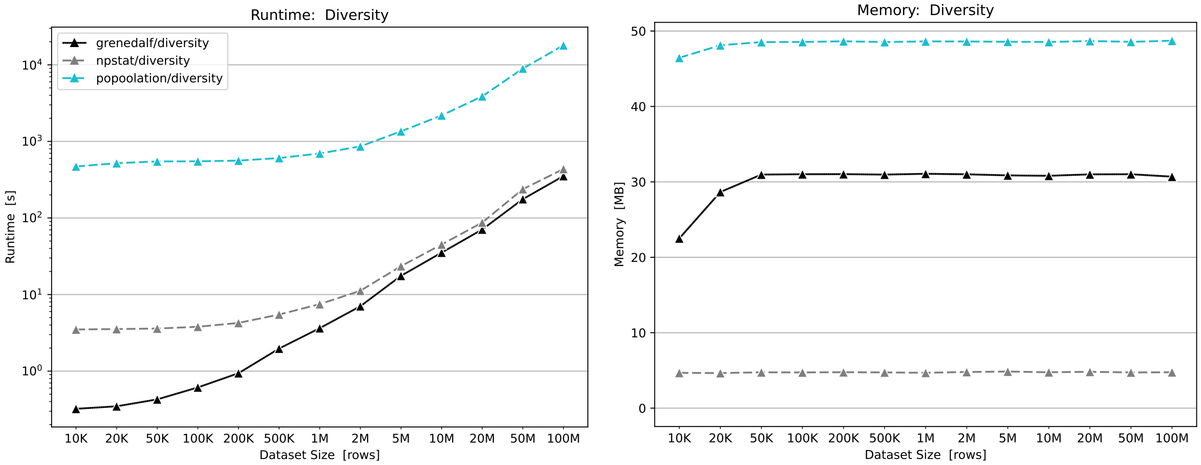

Figure 6: Runtimes (log-log scale) and memory usage (linear–log scale) for computing diversity measures, for dataset sizes of 10 K to 100 M genomic positions, on randomly simulated data.

In order to measure the minimally expected improvements of GRENEDALF over existing tools, we simulated datasets using random read depths and allele counts to create `pileup` test files. The read depth in these files is rather small, only up to 500, so that the computation of the Pool-seq correction terms is not dominating the runtime too much. We hence avoid data-dependent effects here, as for example seen in Figure 4 when hitting genome positions with higher read depth. This hence serves as a benchmark of the expected lower boundary of the speedup of grenedalf compared to other tools, showing the raw processing speed for tasks such as file reading and windowing of the data.

We again computed Tajima's D in windows of 1000 base pairs along the genome. Due to the fixed low read depth, no surge in the runtime is observed here, and instead the overall trend in file processing speed is more evident. At larger sample sizes, GRENEDALF is about 50 times as fast as PoPOOLATION, and 1.2 times as fast as NPSTAT, which is written in `C`, and also comparably efficient, but lacks support for multi-threaded speedups.

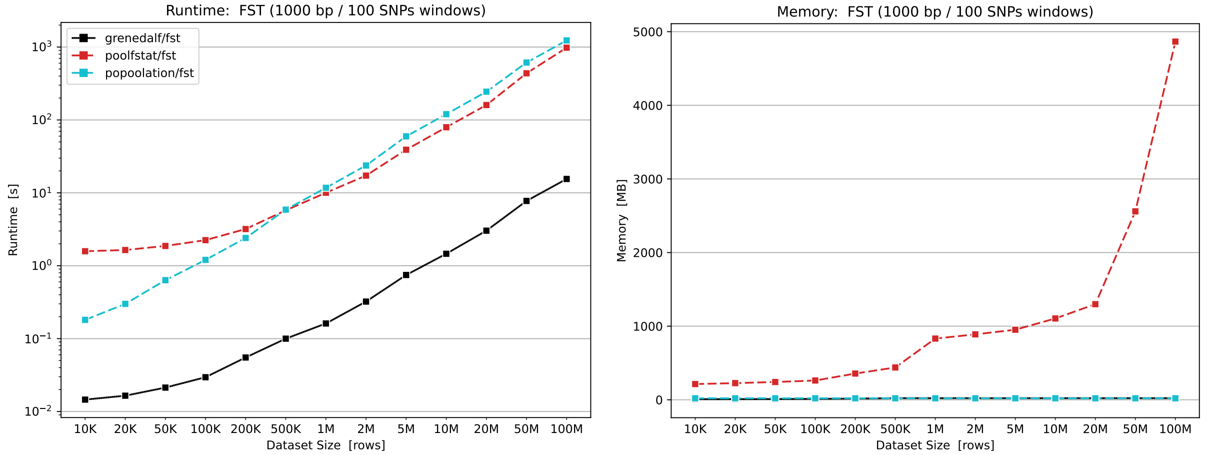

Figure 7: Runtimes (log-log scale) and memory usage (linear–log scale) for computing F_{ST}, for dataset sizes of 10 K to 100 M genomic positions, on randomly simulated data, in windows of 1000 base pairs and 100 SNPs.

For the reasons explained in Figure 6, we here simulated datasets with random (small) read depths and allele counts to create `sync` test files containing two samples. We then computed F_{ST} in windows along the genome. For GRENEDALF and POPOOLATION2, we used windows of 1000 bp. As POOLFSTAT does not seem to support windows of fixed size along the genome, we instead used windows of 100 SNP length; as we simulated with 10% variant positions, this averages out to the same total number of windows being processed. Note that GRENEDALF can also use windows with a fixed number of SNPs (as opposed to windows of a fixed length along the genome), which we omit here for simplicity.

Again, GRENEDALF outperforms both other tools in terms of runtime by a factor of 60 and 80, respectively, for the largest input file. This is the minimally expected speed up, and should be equally as fast or even faster on real world data.

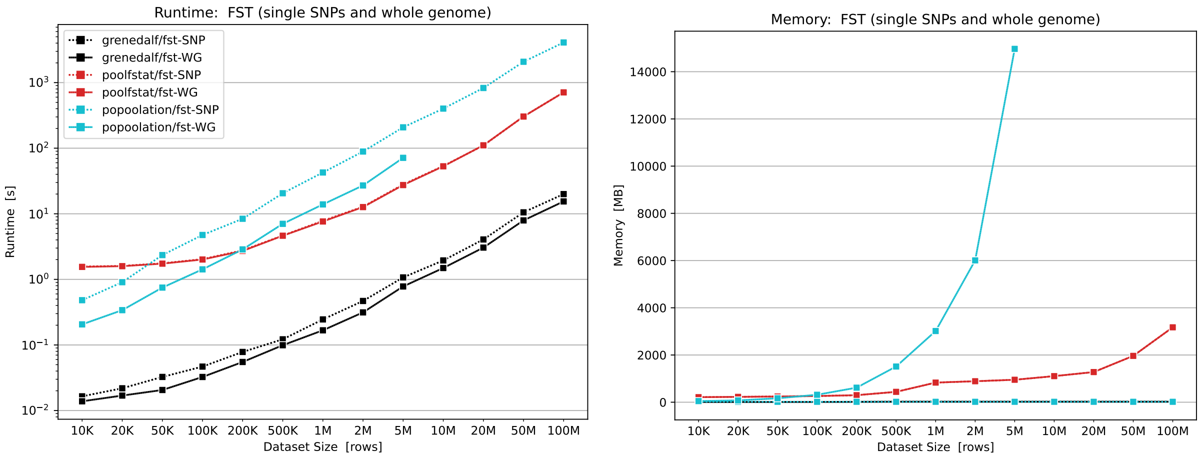

Figure 8: Runtimes (log-log scale) and memory usage (linear–log scale) for computing F_{ST}, for dataset sizes of 10 K to 100 M genomic positions, on randomly simulated data, on single SNPs and the whole genome.
This is the same data and setup as explained in Figure 7, but instead of using sliding windows, we here computed F_{ST} for single SNPs, and for the whole genome, in order to show the effects of these choices. For PoPoolation2, we could not compute whole-genome F_{ST} on larger datasets, as it needs to keep data in memory for the whole window, which becomes prohibitive at full genome scale. Despite also keeping the whole genome in memory, the overall memory requirements for poolfstat are lower, and it hence was able to compute F_{ST} for the large datasets here as well. However, this still can easily become a limitation when computing F_{ST} for more samples than used in this test. The runtime and memory for poolfstat are virtually identical with both types of windowing. Again, grenedalf outperforms both in terms of runtime. We implemented a streaming approach for whole genome data, so that even in that case, our memory requirements are not affected by the genome sizes at all (in fact, for the whole genome we need less memory than for windowed approaches, as we do not even need to keep the data for each window in memory).

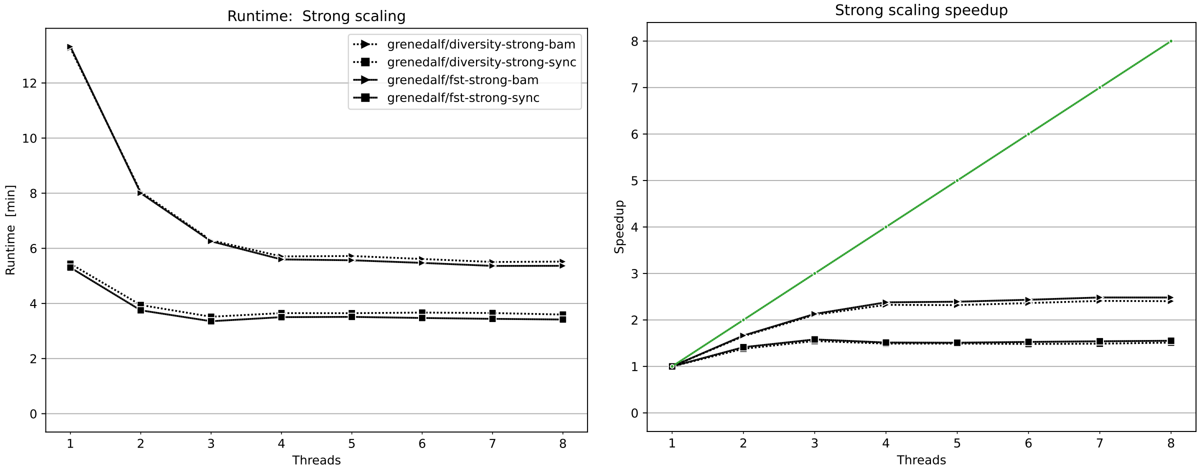

Figure 9: Runtimes (linear scale) and strong scaling speedup for computing Tajima's D and F_{ST} with multiple threads, for real-world data, in windows of 1000 base pairs.

We here again used real world data from our GrENE-net project, in order to test scaling efficiency of GRENEDALF when using multiple cores to speed up the computation. In each test, we used 8 samples, in two different input file formats (`bam` and `sync`), and computed Tajima's D for each sample, as well as F_{ST} between each pair of samples (28 pairs), both for the whole genome. The left hand plot shows the total runtime of each test, with an increasing number of threads, up to the number of cores on the computer where this was tested. The right hand plot shows the strong scaling speedup based on these runtimes, i. e., the runtime with one thread divided by the runtime with multiple threads. The green identity line is the theoretical maximum speedup.

As before, reading from `sync` files is significantly faster than reading from `bam` files, and accounts for a large portion of the runtime. This test however shows that using multiple threads on this relatively small test dataset only initially yields speedup, before levelling off.

This scaling inefficiency is likely due to Amdahl's law: The amount of scaling that can be achieved with multiple threads is limited by the portion of the runtime that is not parallelizable. For datasets with few samples, the parallelizable portion of the program (e. g., the input file reading, and the actual computation of F_{ST}) is relatively small compared to the linear portion (e. g., the stream iteration, synchronizing the positions along the chromosome, and the output writing). Furthermore, with only a few samples, the thread synchronization overhead that becomes necessary when using more than one thread for the computation has a higher impact: Our internal memory layout buffers data and computes statistics per position in the genome. With few samples, the computational work at each position is small, and hence, the synchronization between threads at every position limits the efficiency. Lastly, for the `bam` input, we see an additional inefficiency in the implementation of HTSLIB, which we internally use for reading `bam` files. The majority of the time for these files is spend in the pointer-heavy function to resolve the CIGAR string. This suggests that the reading performance for `bam` files is memory-bound, so that more cores do not yield additional speedups here.

At the time being, we do not consider it worth optimizing this further though. This is because for larger datasets, where more work is done per position (and hence the parallelizable portion of the program is larger), and where runtime starts to matter more, we do see a significant speedup with multiple threads, as shown in Figure 10.

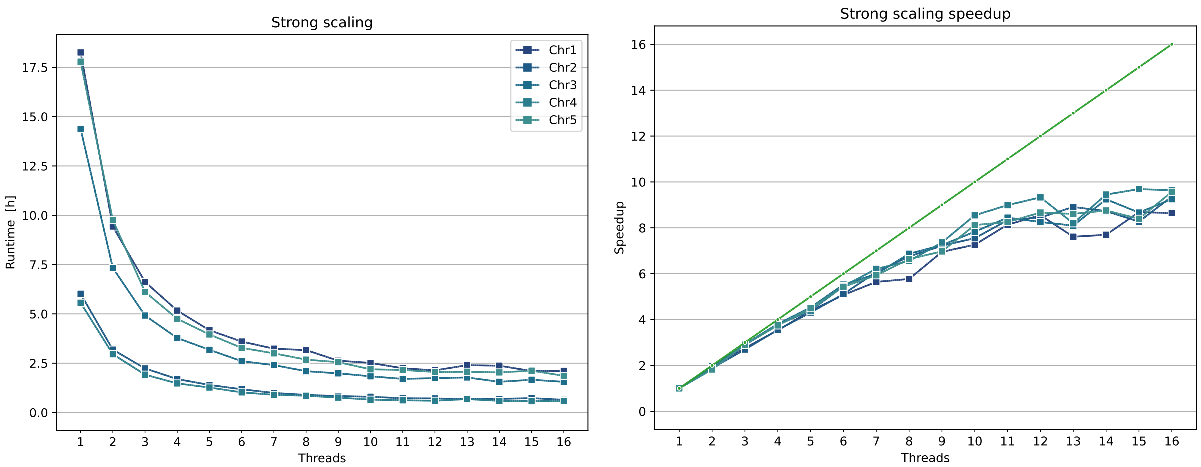

Figure 10: Runtimes (linear scale) and strong scaling speedup for computing pairwise whole-genome F_{ST} with multiple threads, for 2415 samples.

Here, we used the dataset of Figure 2, with 2,415 samples, for a large-scale strong scaling test. We computed pairwise F_{ST} between all 2,914,905 pairs of samples, for the whole genome with 2,948,475 variant positions. As this was run on a computer cluster, we were able to test with up to 16 threads. Each cluster node was allocated with 4 extra cores on top of the cores needed for each computational thread, in order to avoid core over-subscription. To compensate for cluster usage fluctuations, the results shown here are the minimum times of three independent runs for each measurements. We furthermore split the dataset into its 5 chromosomes, in order to get more measurement points, and shorter overall waiting time to run the tests.

The left hand plot shows the total runtimes. In contrast to Figure 9, we do see a significant speedup here for increasing numbers of threads. With this large dataset, the computation of all ≈3 million pairs of F_{ST} constitutes a larger portion of the runtime, meaning that a larger portion of the work is parallelized. Hence, more computational work is being executed per position, also meaning that thread synchronization overhead has less influence here.

The right hand plot again shows the strong scaling speedup, that is, the runtime with one thread divided by the runtime with multiple threads. For up to ≈10 threads, we observe almost ideal speedup (green line), levelling off after that due to Amdahl's law. This is expected, and can be interpreted to indicate that (for this dataset) using more than 10 threads has diminishing returns. We note though that this is likely fast enough for any contemporary dataset: The overall runtime across all 2,914,905 pairs of samples and all five chromosomes using 10 threads was ≈8 h.

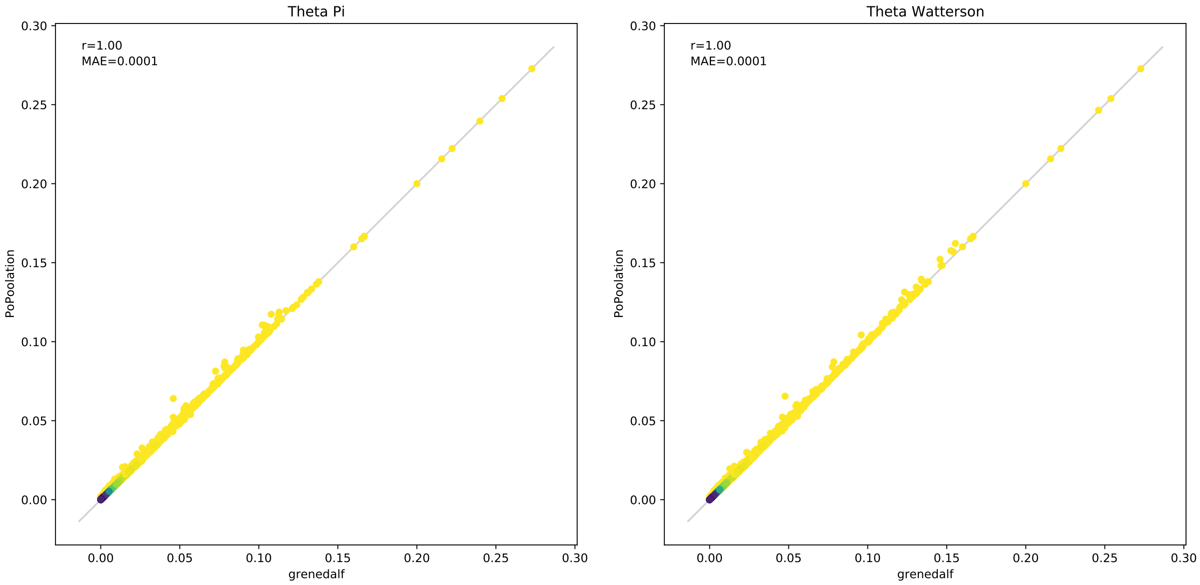

Figure 11: Comparison of grenedalf and PoPoolation for Theta Pi and Theta Watterson on real-world data.
We here compare the results of the implementation in GRENEDALF to those obtained with POPOOLATION for Theta Pi (left) and Theta Watterson (right). The input data is the same as used in Figure 4, namely Pool-Seq samples from our GrENE-net experiment, in order to check for any discrepancies between the tools on real-world data. Such data often contains types of noise and variation that are hard to capture realistically in simulated data. We used a window size of 1000bp, and matching parameters for minimum counts and read depths. The plots compare the values per window obtained with both tools, with the coloring based on a Gaussian kernel density estimate, indicating that most values of the diversity estimators are rather small here.
Our results match closely with those obtained from POPOOLATION. During development of GRENEDALF, we however noted some initial discrepancies between the two tools that resulted from differences in the filtering of positions with a high number of deletions in the data. This was particularly prevalent in windows with a low number of variant positions and low read depths, where omitting a single SNP due to some filter setting already has a large impact on the overall estimate for the window. We have since adapted our filtering of deletions to match more closely with that of POPOOLATION, as seen here. The remaining slight differences between the tools are likely resulting from similar edge cases in the implementation details, or might even be of numerical nature, but are small enough to not warrant further investigation.

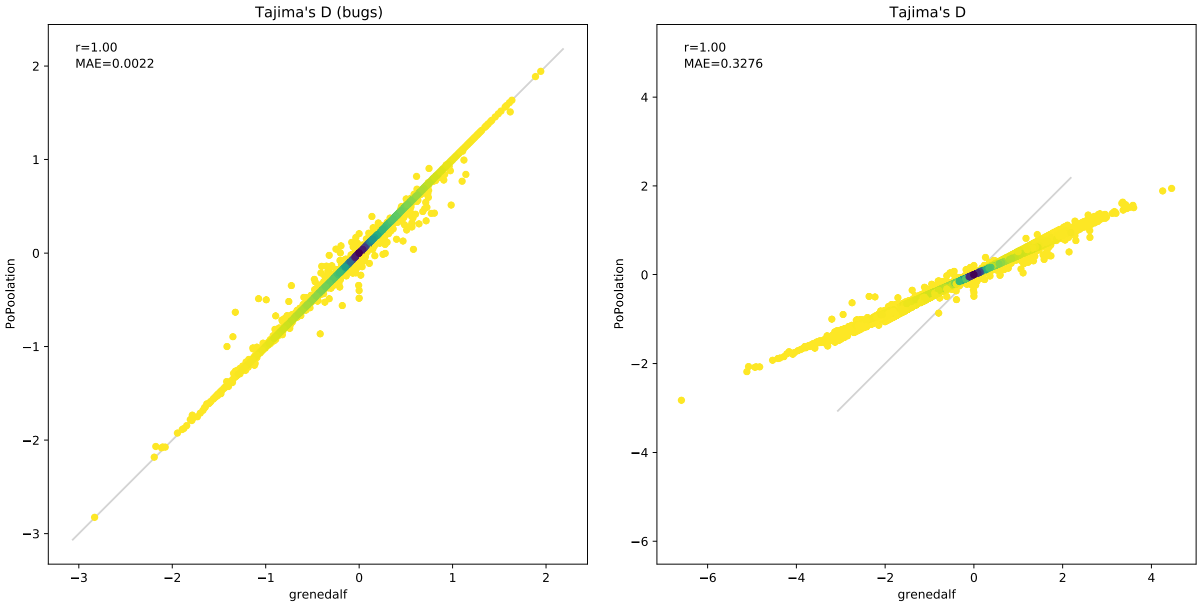

Figure 12: Comparison of grenedalf and PoPoolation for Tajima's D on real-world data. The setup for this figure is equivalent to Figure 11, but shows the comparison for Tajima's D. As explained in our equations document, the implementation of Tajima's D in PoPoolation contains several bugs that affect the results; we hence show the comparison to grenedalf here with our intentional re-implementation of those bugs for purposes of comparison (left). Similar to Figure 11, the results are highly consistent between the tools, with only a few positions not fully matching.
We also show the effect of those bugs (right): At fixed pool sizes with relatively consistent read depth throughout the sample, the bugs act as a constant factor applied to the values. However, as detailed in our equations document, even with those bugs removed, this estimator of Tajima's D does not fully capture the complexity of the data, and is in fact not comparable with the numerical results from a "classical" estimator of Tajima's D based on individual data. In the Pool-seq case, other population characteristics need to be taken into account, which the estimator does not do. We hence recommend to not interpret values obtained from this estimator numerically. For details, see our equations document.

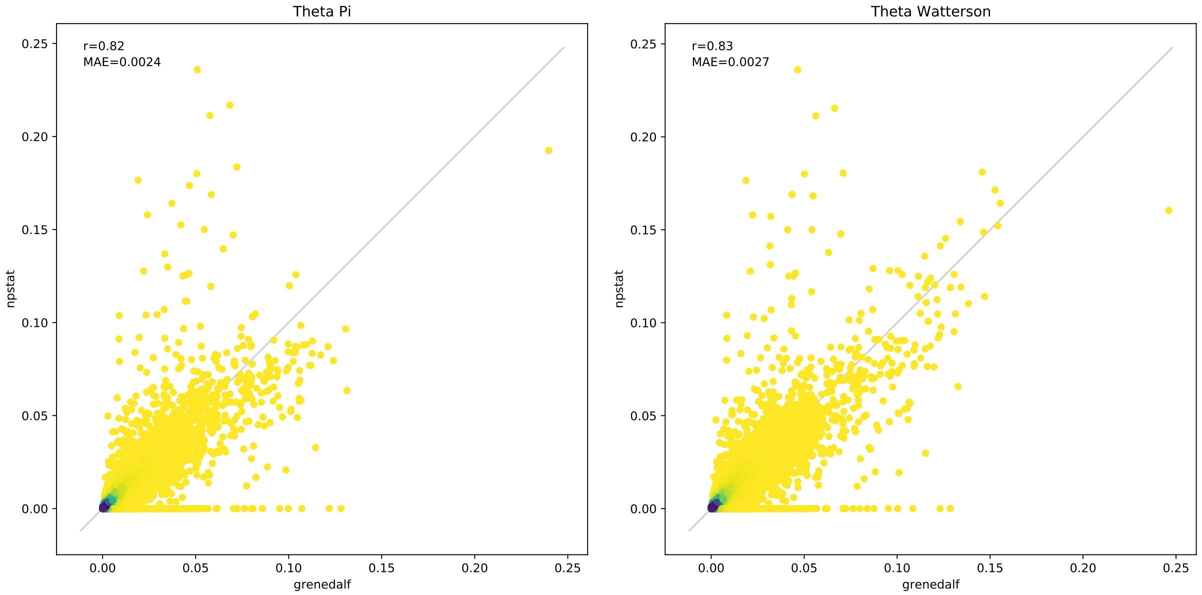

Figure 13: Comparison of grenedalf and npstat for Theta Pi and Theta Watterson on real-world data, with a window size of 1000bp.
This figure is the equivalent of Figure 11 for comparing the results of the implementation in GRENEDALF to those obtained with NPSTAT for Theta Pi (left) and Theta Watterson (right). The difference between the tools is more pronounced here. Based on our inspection, and following the reasoning as outlined in Figure 11, we find that this is due to slight differences in the conditions and order of filters applied to the data, and similar implementation details that can have large effect in windows with few variants and low read depth. We however did not attempt to track down the exact origins of these differences in the source code.
Instead, we compared the values obtained with GRENEDALF and NPSTAT. We find that the differences in values obtained with both tools between the two estimators (Theta Pi and Theta Watterson) are highly consistent (correlation coefficient of 0.98). In other words, positions in the data where GRENEDALF and NPSTAT give different estimates of Theta Pi also give systematically different estimates of Theta Watterson (and vice versa), with the difference between values obtained from both tools at a given position being almost identical for both statistics. This is also visible in the two plots here, where the pattern of scattering between the two tools looks almost identical for both estimators.
This corroborates our finding that the differences between GRENEDALF and NPSTAT are indeed artifacts of filter implementation (or related implementation details such as which positions are counted in the denominators of the estimators), and not in the actual statistics implementations. We further show that this is the case in Figure 14.

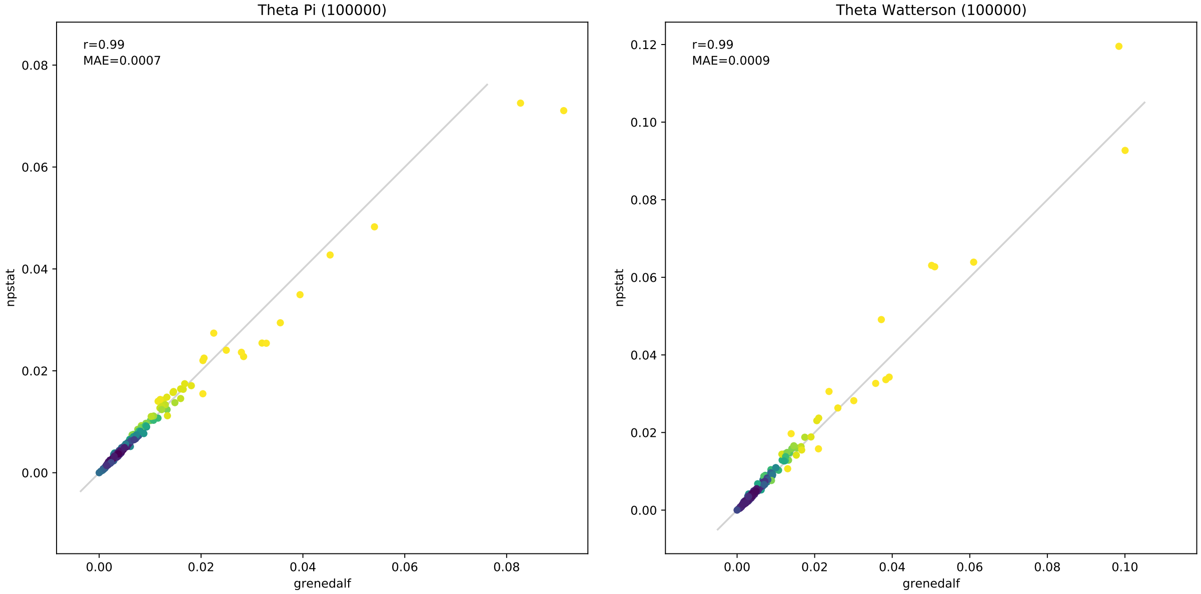

Figure 14: Comparison of grenedalf and npstat for Theta Pi and Theta Watterson on real-world data, with a window size of 100,000bp.
This figure is the equivalent of Figure 13 with a larger window size. As predicted there, we here indeed observe a stronger match between GRENEDALF and NPSTAT. This is because the windows here contain more variants, so that variants that are treated differently between the tools based on slight implementation details do not exert large effects. Thus, differences in values between the tools are likely caused by slight edge cases in the implementation details for small windows with low number of variants and low read depth. We hence do not further investigate this here, as these decisions are to some degree arbitrary, and diminish at larger read depths and in larger windows with more variant positions.
We note that this implies to not over-interpret high values of either statistic in windows with low read depth and low variation: estimates in such windows tend to have high variance, where slight implementation details can have a large effect. Larger window sizes can be used to average out the effect of these outliers.

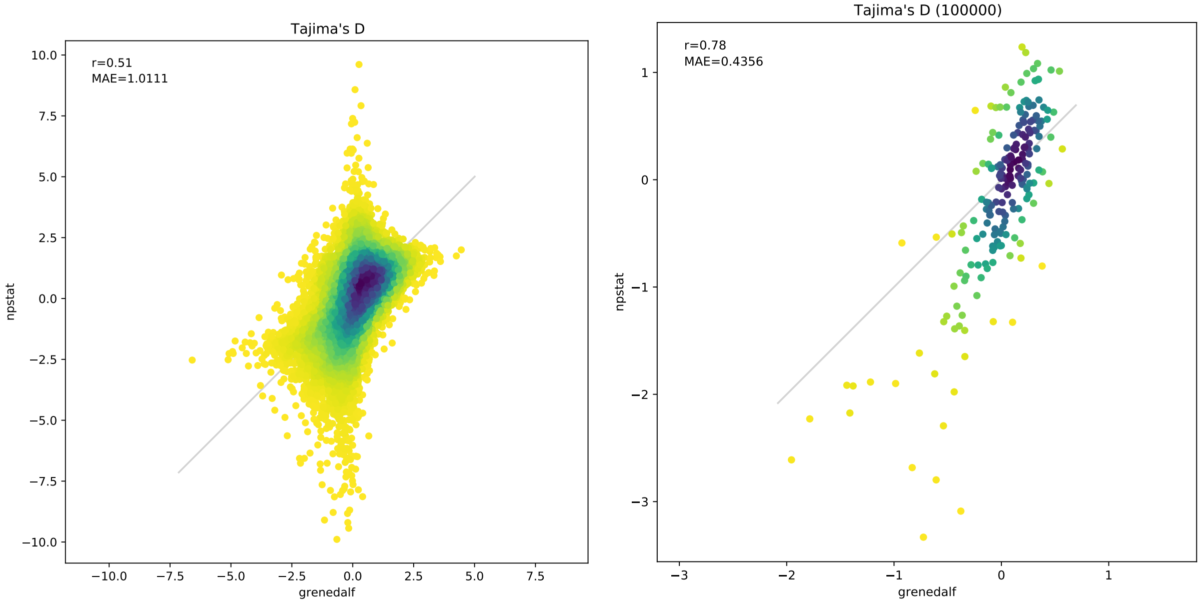

Figure 15: Comparison of grenedalf and npstat for Tajima's D on real-world data. Based on Figure 13 and Figure 14, we here show the comparison between GRENEDALF and NPSTAT for Tajima's D, for both window sizes of 1000bp (left) and 100,000bp (right). The discrepancies between the two tools is most pronounced at the small window size, where no strong correlation is present. Given the results of the previous figures, this is expected, as the discrepancies between the tools are exacerbated when computing the difference between Theta Pi and Theta Watterson in order to get the estimator for Tajima's D. At the larger window size, there is a more significant correlation between the tools, but estimates seem to be stretched by a constant factor in NPSTAT.

Given the difficulties and shortcomings of the estimator of Tajima's D for pool sequencing that we describe in our equations document, we decided to not further investigate this. Likely, the differences seen here are a combination of noise in the data in low read depth conditions, amplified by slight implementation differences, and further obscured by statistical deficits of the estimator itself. We hence urge practitioners to exercise caution when interpreting values of Tajima's D numerically.

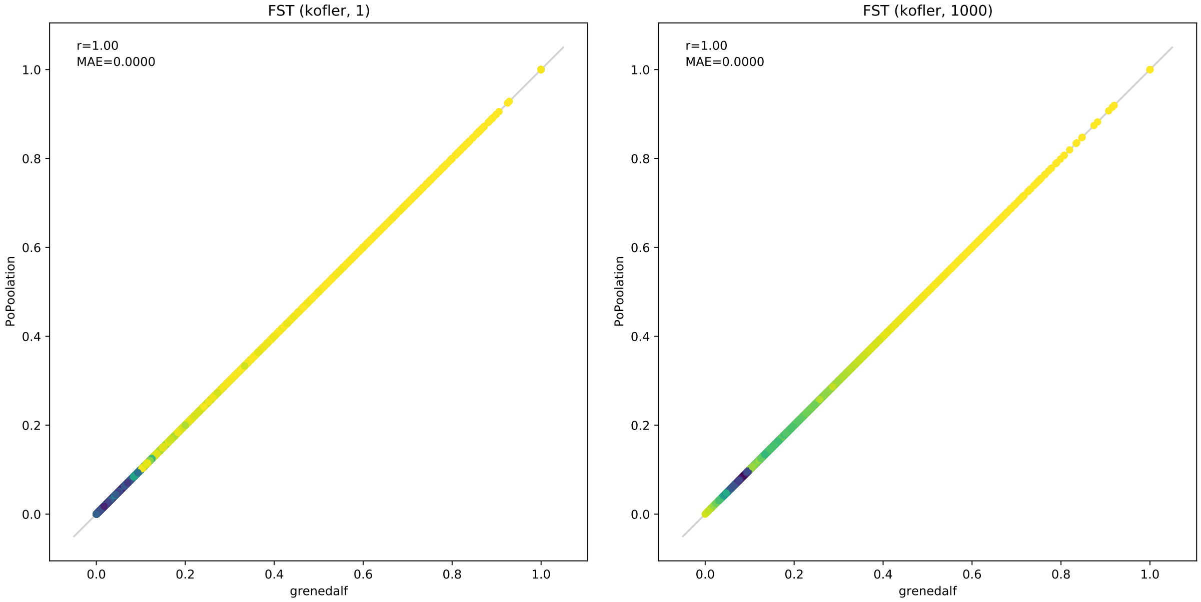

Figure 16: Comparison of grenedalf and PoPoolation2 of values for F_{ST} Kofler on real-world data.
We here compare the results of the implementation of the "Kofler" estimator of F_{ST} in GRENEDALF to those obtained with PoPOOLATION2 for different window sizes. The input data is the same as used in Figure 5, and as explained in the plots above. We ran both tools with window sizes of 1bps and 1000bps, respectively, in order to evaluate potential single SNP differences as well as window normalization differences. Other parameters such as minimum counts and read depths were matching otherwise. The plots show the values per window obtained with each tool, with coloring being based on a Gaussian kernel density estimate. Both tools produce almost identical results.

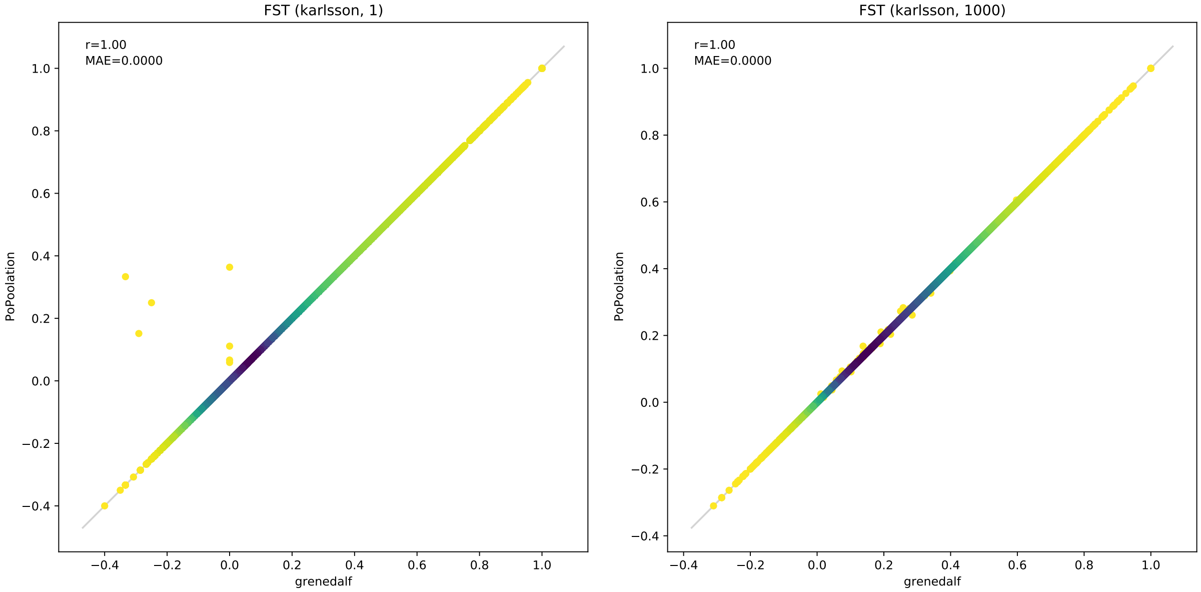

Figure 17: Comparison of grenedalf and PoPoolation2 of values for F_{ST} Karlsson on real-world data.
This figure is the equivalent of Figure 16 for the "Karlsson" estimator of F_{ST}. The setup, results, and conclusions are exactly the same; see there for details. Note that due to the corrections applied in the estimator, negative values of F_{ST} can be observed here. This is expected; see our equation document for details.

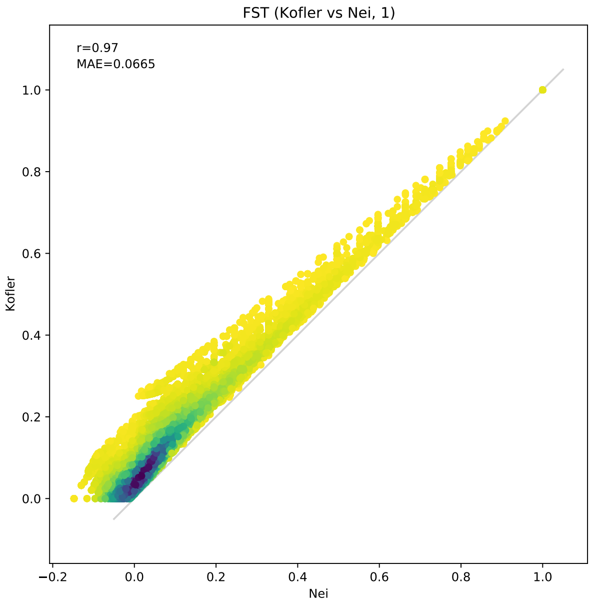

Figure 18: Comparison of the Kofler vs Nei estimators of F_{ST} on real-world data. As shown in our assessment of the Pool-seq correction equations for F_{ST}, the estimator as implemented in PoPoolation2, which we call the "Kofler" estimator, is biased upward for small pool sizes and small read depths. Here, we show the effect of this bias, compared to our asymptotically unbiased estimator of Nei's definition of F_{ST}, on single SNPs and in windows of 1000bps. The data again consists of two samples from GrENE-net, as introduced above. All computations where conducted with grenedalf. To focus on the effect of read depth, we used a pool size of 100 here (although in reality, the data came from a smaller pool of individuals). See also Figure 19.
It is evident that the Kofler estimator has consistently larger values, independent of the window size. This corroborates our theoretical findings of the estimator being upwards biased, as explained in our equations document.

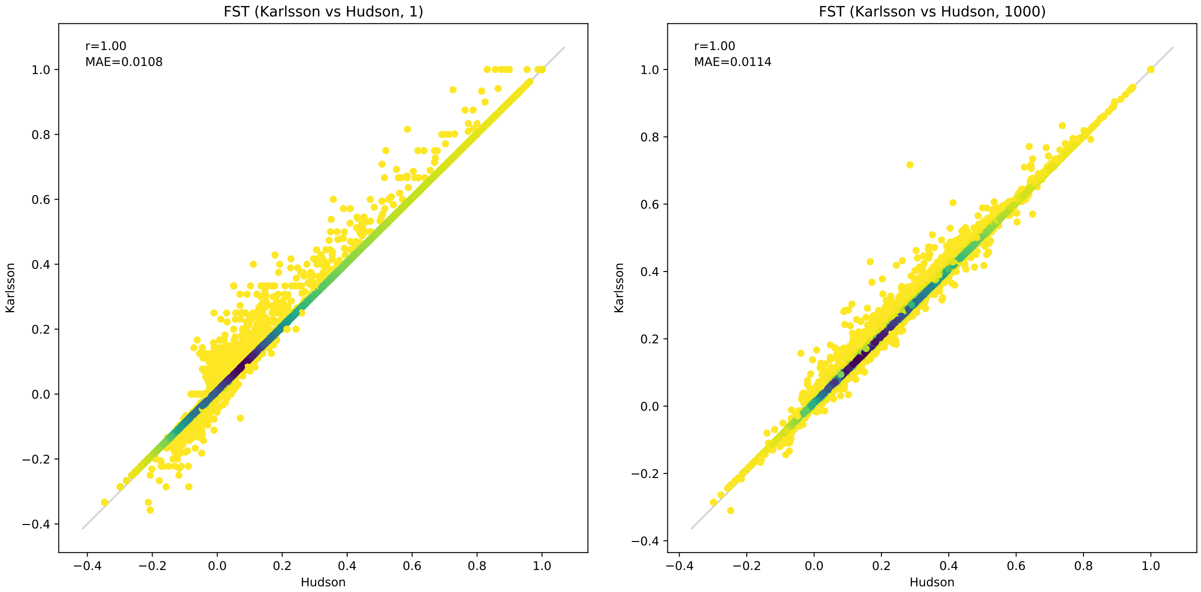

Figure 19: Comparison of the Karlsson vs Hudson estimators of F_{ST} on real-world data. Similar to Figure 18, here we evaluate the differences of the "Karlsson" estimator compared to our asymptotically unbiased estimator following Hudson's definition of F_{ST}, on single SNPs and in windows of 1000bp. We again used a pool size of 100 here. As the Karlsson estimator is only biased for low pool sizes, it behaves virtually identical to our Hudson estimator at that pool size, as also shown in the equations assessment. The scattering seen in the plots here solely come from multiallelic positions, where the third and forth allele are taken into account by the Hudson estimator, but ignored by the Karlsson estimator (as a consequence of it being defined for biallelic SNPs). The differences completely disappear when additionally filtering the input data for biallelic SNPs or constraining the data by removing the counts of the third and fourth alleles.

4 References

[1] Czech L, Barbera P, Stamatakis A (2020) Genesis and Gappa: processing, analyzing and visualizing phylogenetic (placement) data. *Bioinformatics* 36(10):3263–3265.

[2] Kofler R, et al. (2011) PoPoolation: A Toolbox for Population Genetic Analysis of Next Generation Sequencing Data from Pooled Individuals. *PLoS ONE* 6(1):e15925.

[3] Kofler R, Pandey RV, Schlotterer C (2011) PoPoolation2: identifying differentiation between populations using sequencing of pooled DNA samples (Pool-Seq). *Bioinformatics* 27(24):3435–3436.

[4] Hivert V, Leblois R, Petit EJ, Gautier M, Vitalis R (2018) Measuring Genetic Differentiation from Pool-seq Data. *Genetics* 210(1):315–330.

[5] Gautier M, Vitalis R, Flori L, Estoup A (2022) f-statistics estimation and admixture graph construction with Pool-Seq or allele count data using the R package poolfstat. *Mol. Ecol. Resour.* 22(4):1394–1416.

[6] Ferretti L, Ramos-Onsins SE, Pérez-Enciso M (2013) Population genomics from pool sequencing. *Molecular Ecology* 22(22):5561–5576.

[7] Li H, et al. (2009) The Sequence Alignment/Map format and SAMtools. *Bioinformatics* 25(16):2078–2079.

[8] Bonfield JK, et al. (2021) HTSlib: C library for reading/writing high-throughput sequencing data. *Gigascience* 10(2):1–6.

[9] Danecek P, et al. (2011) The variant call format and VCFtools. *Bioinformatics* 27(15):2156–2158.

[10] Tilk S, et al. (2019) Accurate allele frequencies from ultra-low coverage Pool-Seq samples in Evolve-and-Resequence experiments. *G3 Genes Genomes Genetics* 9(12):4159–4168.

[11] Kessner D, Turner TL, Novembre J (2013) Maximum likelihood estimation of frequencies of known haplotypes from pooled sequence data. *Mol. Biol. Evol.* 30(5):1145–1158.

[12] Czech L, et al. (2022) Monitoring rapid evolution of plant populations at scale with pool-sequencing. *bioRxiv.*

[13] Czech L, Exposito-Alonso M (2022) grenepipe: A flexible, scalable, and reproducible pipeline to automate variant calling from sequence reads. *Bioinformatics.*
